# Author Correction: Genome-wide association study of alcohol consumption and use disorder in 274,424 individuals from multiple populations

**DOI:** 10.1038/s41467-019-10254-5

**Published:** 2019-05-17

**Authors:** Henry R. Kranzler, Hang Zhou, Rachel L. Kember, Rachel Vickers Smith, Amy C. Justice, Scott Damrauer, Philip S. Tsao, Derek Klarin, Aris Baras, Jeffrey Reid, John Overton, Daniel J. Rader, Zhongshan Cheng, Janet P. Tate, William C. Becker, John Concato, Ke Xu, Renato Polimanti, Hongyu Zhao, Joel Gelernter

**Affiliations:** 1 University of Pennsylvania Perelman School of Medicine, Philadelphia, PA 19104 USA; 2 Crescenz Veterans Affairs Medical Center, Philadelphia, PA 19104 USA; 3 Yale School of Medicine, New Haven, CT 06511 USA; 4 Veterans Affairs Connecticut Healthcare System, West Haven, CT 06516 USA; 5 University of Louisville School of Nursing, Louisville, KY 40202 USA; 6 Yale School of Public Health, New Haven, CT 06511 USA; 7 VA Palo Alto Health Care System, Palo Alto, CA 94304 USA; 8 Stanford University School of Medicine, Stanford, CA 94305 USA; 9 Massachusetts General Hospital, Harvard Medical School, Boston, MA 02114 USA; 10 Regeneron Genetics Center, Tarrytown, NY 10591 USA

**Keywords:** Genome-wide association studies, Addiction, Epidemiology, Genetics research

Correction to: *Nature Communications* 10.1038/s41467-019-09480-8, published online 2 April 2019

The original version of this Article omitted the following from the Acknowledgements: ‘Supported by the Mental Illness Research, Education and Clinical Center of the Veterans Integrated Service Network 4 of the Department of Veterans Affairs.’ This has now been corrected in both the PDF and HTML versions of the Article.

