# Author Correction: Genome-wide association study of alcohol consumption and use disorder in 274,424 individuals from multiple populations

**DOI:** 10.1038/s41467-019-11916-0

**Published:** 2019-09-03

**Authors:** Henry R. Kranzler, Hang Zhou, Rachel L. Kember, Rachel Vickers Smith, Amy C. Justice, Scott Damrauer, Philip S. Tsao, Derek Klarin, Aris Baras, Jeffrey Reid, John Overton, Daniel J. Rader, Zhongshan Cheng, Janet P. Tate, William C. Becker, John Concato, Ke Xu, Renato Polimanti, Hongyu Zhao, Joel Gelernter

**Affiliations:** 1 University of Pennsylvania Perelman School of Medicine, Philadelphia, PA 19104 USA; 2 Crescenz Veterans Affairs Medical Center, Philadelphia, PA 19104 USA; 3 Yale School of Medicine, New Haven, CT 06511 USA; 4 Veterans Affairs Connecticut Healthcare System, West Haven, CT 06516 USA; 5 University of Louisville School of Nursing, Louisville, KY 40202 USA; 6 Yale School of Public Health, New Haven, CT 06511 USA; 7 VA Palo Alto Health Care System, Palo Alto, CA 94304 USA; 8 Stanford University School of Medicine, Stanford, CA 94305 USA; 9 Massachusetts General Hospital, Harvard Medical School, Boston, MA 02114 USA; 10 Regeneron Genetics Center, Tarrytown, NY 10591 USA

**Keywords:** Genome-wide association studies, Addiction, Epidemiology, Genetics research

Correction to: *Nature Communications* 10.1038/s41467-019-09480-8, published online 2 April 2019.

In the original version of this Article, several numbers were given incorrectly in Tables 1 and 2 in columns 5 (EAF), 6 (*N*) and 7 (*Z*-score). The mistakes arose when one population was removed from the analyses but the numbers were not updated in the tables. These have now been corrected in both the HTML and PDF versions of the Article. The incorrect versions of these tables appear below as Tables 1 and 2 and the correct versions as Tables 3 and 4, respectively.

**Table 1:** Genome-wide significant associations for AUDIT-C in the trans-population meta-analysis

rsID	Chr:pos^a^	A1/A2	Gene^b^	EAF	*N*	*Z*-score	*P*_EA	*P*_AA	*P*_LA	*P*_EAA	*P*_SAA	Effect	*P*_ meta
rs1260326	2:27730940	C/T	*GCKR* ^c^	0.652	270,226	8.22	1.74 × 10^−16^	0.110	0.067	0.987	0.739	+++++	2.04 × 10^−16^
rs2683616	2:58035555	A/G	*VRK2* ^d^	0.624	211,399	6.22	1.80 × 10^−9^	NA	0.060	0.487	NA	+?+−?	4.95 × 10^−10^
rs12639940	4:39420981	A/G	*KLB* ^c^	0.613	194,761	5.93	3.45 × 10^−9^	NA	NA	0.626	NA	+??+?	3.06 × 10^−9^
rs1229984	4:100239319	C/T	*ADH1B* ^c^	0.970	272,358	24.56	4.83 × 10^−102^	1.31 × 10^−19^	4.40 × 10^−16^	9.05 × 10^−3^	NA	++++?	3.62 × 10^−133^
rs142783062	4:100270960	D/I	*ADH1C* ^c^	0.345	271,444	9.82	2.04 × 10^−14^	4.75 × 10^−7^	4.90 × 10^−4^	0.019	0.779	+++++	9.50 × 10^−23^
rs13107325	4:103188709	C/T	*SLC39A8* ^c^	0.937	270,248	11.45	1.43 × 10^−25^	1.07 × 10^−4^	2.23 × 10^−3^	NA	NA	+++??	2.24 × 10^−30^
rs4423856	4:150984857	T/C	*DCLK2* ^d^	0.796	212,444	5.66	3.60 × 10^−8^	NA	0.289	0.574	0.144	+?+++	1.48 × 10^−8^
rs2961816	5:50443691	A/C	*ISL1* ^d^	0.683	260,828	5.74	1.24 × 10^−7^	0.021	0.641	0.932	0.137	+++++	9.75 × 10^−9^
rs4841132	8:9183596	A/G	*PPP1R3B* ^d^	0.101	276,763	5.46	2.75 × 10^−6^	6.59 × 10^−3^	0.226	NA	0.148	−−−?+	3.62 × 10^−8^
rs62033408	16:53827962	A/G	*FTO* ^c^	0.678	270,067	9.08	2.20 × 10^−15^	4.78 × 10^−5^	0.229	0.177	0.027	+++++	1.11 × 10^−19^
rs9902512	17:47094274	C/G	*IGF2BP1* ^c^	0.664	207,229	-5.81	3.81 × 10^−8^	NA	0.055	0.782	NA	−?−−?	6.24 × 10^−9^
rs142997686	17:79419159	D/I	*BAHCC1* ^c^	0.384	211,314	5.84	1.77 × 10^−9^	NA	0.944	0.434	0.840	+?−+−	5.39 × 10^−9^
rs75723348	22:41420679	T/G	*RBX1* ^d^	0.736	275,328	5.55	2.97 × 10^−7^	0.063	0.072	0.536	0.579	+++++	1.11 × 10^−8^

The loci shown represent completely independent signals after conditioning analyses

*A1* effect allele, *A2* other allele, *EAF* effective allele frequency, *EA* European American, *AA* African American, *LA* Latino American, *EAA* East Asian American, *SAA* South Asian American

^a^Human Genome hg19 assembly

^b^Gene nearest to the lead SNP

^c^Protein-coding gene contains the lead SNP

^d^Protein-coding gene nearest to the lead SNP

**Table 2:** Genome-wide significant associations for AUD in the trans-population meta-analysis

rsID	Chr:pos^a^	A1/A2	Gene^b^	EAF	*N*	*Z*-score	*P*_EA	*P*_AA	*P*_LA	*P*_EAA	*P*_SAA	Effect	*P*_ meta
rs1260326	2:27730940	C/T	*GCKR* ^c^	0.651	271,763	7.33	1.44 × 10^−16^	0.679	0.778	0.830	0.820	+++−+	2.27 × 10^−13^
rs540606	2:45138507	A/G	*SIX3* ^d^	0.409	213,336	-6.49	2.84 × 10^−10^	NA	0.175	0.411	NA	−?−−?	8.58 × 10^−11^
rs5860563	4:100047157	D/I	*ADH4* ^c^	0.722	277,270	-6.21	7.63 × 10^−5^	9.85 × 10^−7^	0.035	NA	0.412	−−−?+	1.12 × 10^−9^
rs1229984	4:100239319	C/T	*ADH1B* ^c^	0.969	273,904	19.54	4.51 × 10^−74^	4.18 × 10^−5^	5.81 × 10^−17^	0.032	NA	++++?	4.68 × 10^−85^
rs1612735	4:100258007	T/C	*ADH1C* ^c^	0.656	271,471	-8.86	1.75 × 10^−14^	6.42 × 10^−5^	0.022	0.938	0.054	−−−++	7.90 × 10^−19^
rs13107325	4:103188709	C/T	*SLC39A8* ^c^	0.937	271,784	7.60	2.73 × 10^−14^	0.064	0.363	NA	NA	+++??	2.97 × 10^−14^
rs7906104	10:110497101	T/C		0.271	275,977	-5.98	3.15 × 10^−7^	8.72 × 10^−3^	0.357	0.195	0.106	−−−−−	3.17 × 10^−9^
rs61902812	11:113374420	A/C	*DRD2* ^d^	0.306	276,977	-5.59	4.99 × 10^−6^	0.025	0.015	0.220	0.931	−−−−−	2.44 × 10^−8^
rs4936277^e^	11:113431960	A/G	*DRD2* ^d^	0.599	274,128	7.44	2.85 × 10^−11^	0.073	4.36 × 10^−4^	0.200	0.357	+++++	1.01 × 10^−13^
rs1421085	16:53800954	T/C	*FTO* ^c^	0.670	274,340	6.69	3.26 × 10^−10^	0.024	0.525	0.332	0.018	+++++	2.17 × 10^−11^

The loci shown represent completely independent signals after conditioning analyses

*A1* effect allele, *A2* other allele, *EAF* effective allele frequency, *EA* European American, *AA* African American, *LA* Latino American, *EAA* East Asian American, *SAA* South Asian Americans

^a^Human Genome hg19 assembly

^b^Gene nearest to the lead SNP

^c^Protein-coding gene contains the lead SNP

^d^Protein-coding gene nearest to the lead SNP

^e^Different signal than rs61902812

**Table 3:** Genome-wide significant associations for AUDIT-C in the trans-population meta-analysis

rsID	Chr:pos^a^	A1/A2	Gene^b^	EAF	*N*	*Z*-score	*P*_EA	*P*_AA	*P*_LA	*P*_EAA	*P*_SAA	Effect	*P*_ meta
rs1260326	2:27730940	C/T	*GCKR* ^c^	0.652	270,226	8.22	1.74 × 10^−16^	0.110	0.067	0.987	0.739	+++++	2.04 × 10^−16^
rs2683616	2:58035555	A/G	*VRK2* ^d^	0.624	211,399	6.22	1.80 × 10^−9^	NA	0.060	0.487	NA	+?+-?	4.95 × 10^−10^
rs12639940	4:39420981	A/G	*KLB* ^c^	0.613	194,761	5.93	3.45 × 10^−9^	NA	NA	0.626	NA	+??+?	3.06 × 10^−9^
rs1229984	4:100239319	C/T	*ADH1B* ^c^	0.970	272,358	24.56	4.83 × 10^−102^	1.31 × 10^−19^	4.40 × 10^−16^	9.05 × 10^−3^	NA	++++?	3.62 × 10^−133^
rs142783062	4:100270960	D/I	*ADH1C* ^c^	0.345	271,444	9.82	2.04 × 10^−14^	4.75 × 10^−7^	4.90 × 10^−4^	0.019	0.779	+++++	9.50 × 10^−23^
rs13107325	4:103188709	C/T	*SLC39A8* ^c^	0.937	270,248	11.45	1.43 × 10^−25^	1.07 × 10^−4^	2.23 × 10^−3^	NA	NA	+++??	2.24 × 10^−30^
rs4423856	4:150984857	T/C	*DCLK2* ^d^	0.796	212,444	5.66	3.60 × 10^−8^	NA	0.289	0.574	0.144	+?+++	1.48 × 10^−8^
rs2961816	5:50443691	A/C	*ISL1* ^d^	0.683	260,828	5.74	1.24 × 10^−7^	0.021	0.641	0.932	0.137	+++++	9.75 × 10^−9^
rs4841132	8:9183596	A/G	*PPP1R3B* ^d^	0.101	271,192	−5.51	2.75 × 10^−6^	6.59 × 10^−3^	0.226	NA	0.148	---?+	3.62 × 10^−8^
rs62033408	16:53827962	A/G	*FTO* ^c^	0.678	270,067	9.08	2.20 × 10^−15^	4.78 × 10^−5^	0.229	0.177	0.027	+++++	1.11 × 10^−19^
rs9902512	17:47094274	C/G	*IGF2BP1* ^c^	0.664	207,229	−5.81	3.81 × 10^−8^	NA	0.055	0.782	NA	-?--?	6.24 × 10^−9^
rs142997686	17:79419159	D/I	*BAHCC1* ^c^	0.384	211,314	5.84	1.77 × 10^−9^	NA	0.944	0.434	0.840	+?-+-	5.39 × 10^−9^
rs75723348	22:41420679	T/G	*RBX1* ^d^	0.736	269,785	5.71	2.97 × 10^−7^	0.063	0.072	0.536	0.579	+++++	1.11 × 10^−8^

The loci shown represent completely independent signals after conditioning analyses.

*A1* effect allele, *A2* other allele, *EAF* effective allele frequency, *EA* European American, *AA* African American, *LA* Latino American, *EAA* East Asian American, *SAA* South Asian American

^a^Human Genome hg19 assemblyGenome Browser - hg19 assembly

^b^Gene nearest to the lead SNP

^c^Protein-coding gene contains the lead SNP

^d^Protein-coding gene nearest to the lead SNP

**Table 4:** Genome-wide significant associations for AUD in the trans-population meta-analysis

rsID	Chr:pos^a^	A1/A2	Gene^b^	EAF	*N*	*Z*-score	*P*_EA	*P*_AA	*P*_LA	*P*_EAA	*P*_SAA	Effect	*P*_ meta
rs1260326	2:27730940	C/T	*GCKR* ^c^	0.651	271,763	7.33	1.44 × 10^−16^	0.679	0.778	0.830	0.820	+++-+	2.27 × 10^−13^
rs540606	2:45138507	A/G	*SIX3* ^d^	0.409	213,336	−6.49	2.84 × 10^−10^	NA	0.175	0.411	NA	-?--?	8.58 × 10^−11^
rs5860563	4:100047157	D/I	*ADH4* ^c^	0.723	271,487	−6.09	7.63 × 10^−5^	9.85 × 10^−7^	0.035	NA	0.412	---?+	1.12 × 10^−9^
rs1229984	4:100239319	C/T	*ADH1B* ^c^	0.969	273,904	19.54	4.51 × 10^−74^	4.18 × 10^−5^	5.81 × 10^−17^	0.032	NA	++++?	4.68 × 10^−85^
rs1612735	4:100258007	T/C	*ADH1C* ^c^	0.656	271,471	−8.86	1.75 × 10^−14^	6.42 × 10^−5^	0.022	0.938	0.054	---++	7.90 × 10^−19^
rs13107325	4:103188709	C/T	*SLC39A8* ^c^	0.937	271,784	7.60	2.73 × 10^−14^	0.064	0.363	NA	NA	+++??	2.97 × 10^−14^
rs7906104	10:110497101	T/C		0.272	270,278	−5.92	3.15 × 10^−7^	8.72 × 10^−3^	0.357	0.195	0.106	-----	3.17 × 10^−9^
rs61902812	11:113374420	A/C	*DRD2* ^d^	0.304	271,218	−5.58	4.99 × 10^−6^	0.025	0.015	0.220	0.931	-----	2.44 × 10^−8^
rs4936277^e^	11:113431960	A/G	*DRD2* ^d^	0.599	274,128	7.44	2.85 × 10^−11^	0.073	4.36 × 10^−4^	0.200	0.357	+++++	1.01 × 10^−13^
rs1421085	16:53800954	T/C	*FTO* ^c^	0.670	274,340	6.69	3.26 × 10^−10^	0.024	0.525	0.332	0.018	+++++	2.17 × 10^−11^

The loci shown represent completely independent signals after conditioning analyses.

*A1* effect allele, *A2* other allele, *EAF* effective allele frequency, *EA* European American, *AA* African American, *LA* Latino American, *EAA*

East Asian American, *SAA* South Asian Americans

^a^Human Genome hg19 assembly

^b^Gene nearest to the lead SNP

^c^Protein-coding gene contains the lead SNP

^d^Protein-coding gene nearest to the lead SNP

^e^Different signal than rs61902812

