# Supplementary material for: Genome-wide association study of alcohol consumption and use disorder in 274,424 individuals from multiple populations
Source: Nat Commun. 2019 Apr 2;10:1499. doi: 10.1038/s41467-019-09480-8 (PMC6445072; doi:10.1038/s41467-019-09480-8)
Supplement: Supplementary file 1 — Supplementary Information [file 41467_2019_9480_MOESM1_ESM.pdf]

Genome-wide Association Study of Alcohol Consumption and Use Disorder in 274,424 Individuals from Multiple Populations

Kranzler et al.

Supplementary Information

Supplementary Table 1. Demographic characteristics of the samples for GWAS and meta-analyses.

Populations		European American (EA)		African American (AA)		Latino American (LA)		East Asian American (EAA)		South Asian American (SAA)		Trans-population meta	
**Traits**	**Samples**	**N**	**age (mean±SD)**	**N**	**age (mean±SD)**	**N**	**age (mean±SD)**	**N**	**age (mean±SD)**	**N**	**age (mean±SD)**	**N**	**age (mean±SD)**
	all	200,680	63.2 ± 13.0	56,495	57.5 ± 11.8	14,112	55.2 ± 14.9	1,366	51.7 ± 17.6	189	45.3 ± 11.1	272,842	61.5 ± 13.2
**AUDIT-C**	male	186,143	64.0 ± 12.6	49,269	58.8 ± 11.4	12,940	56.2 ± 14.7	1,278	52.1 ± 17.8	NA	NA	249,630	62.5 ± 12.8
	female	14,537	52.4 ± 13.4	7,226	48.7 ± 11.1	1,172	44.2 ± 13.0	NA	NA	NA	NA	22,935	50.8 ± 12.9
	all	202,004	63.3 ± 13.2	56,648	57.6 ± 11.9	14,175	55.3 ± 15.1	1,374	52.0 ± 17.8	190	45.2 ± 14.2	274,391	61.6 ± 13.4
	- cases	34,658	58.3 ± 12.1	17,267	56.9 ± 9.5	3,449	53.7 ± 13.4	164	47.0 ± 17.2	46	45.6 ± 12.7	55,584	57.5 ± 11.5
	- controls	167,346	64.4. ± 13.1	39,381	57.9 ± 12.8	10,726	55.8 ± 15.5	1,210	52.6 ± 18.1	144	45.1 ± 14.7	218,807	62.7 ± 13.6
	male	187,366	64.2 ± 12.7	49,411	58.9 ± 11.5	12,992	56.3 ± 14.8	1,286	52.4 ± 18.1	NA	NA	251,055	62.7 ± 13.0
**AUD**	- cases	32,801	58.8 ± 11.9	16,270	57.3 ± 9.2	3,305	54.2 ± 13.2	151	47.0 ± 14.5	NA	NA	52,527	58.0 ± 11.3
	- controls	154,565	65.3 ± 12.6	33,141	59.6 ± 12.3	9,687	57.0 ± 15.3	1,135	53.1 ± 18.4	NA	NA	198,528	63.9 ± 13.1
	female	14,638	52.5 ± 13.7	7,237	48.7 ± 11.2	1,183	44.3 ± 13.2	NA	NA	NA	NA	23,058	50.9 ± 13.1
	- cases	1,857	49.5 ± 11.6	997	49.1 ± 10.3	144	43.1 ± 11.7	NA	NA	NA	NA	2,998	49.0 ± 11.2
	- controls	12,781	53.0 ± 13.9	6,240	48.6 ± 11.3	1,039	44.5 ± 13.4	NA	NA	NA	NA	20,060	51.2 ± 13.4

NA = not applicable

Supplementary Table 2. ICD-9/10 Codes Used to Define Alcohol Use Disorder.

ICD Code	ICD Description
ICD-9 Alcohol Use Disorder	
305	Nondependent alcohol abuse, unspecified
305.01	Nondependent alcohol abuse, continuous
305.02	Nondependent alcohol abuse, episodic
305.03	Nondependent alcohol abuse, in remission
303	Acute alcoholic intoxication, unspecified
303.01	Acute alcoholic intoxication, continuous
303.02	Acute alcoholic intoxication, episodic
303.03	Acute alcoholic intoxication, in remission
303.9	Other/unspecified alcohol dependence, unspecified
303.91	Other/unspecified alcohol dependence, continuous
303.92	Other/unspecified alcohol dependence, episodic
303.93	Other/unspecified alcohol dependence, in remission
ICD-10 Alcohol Use Disorder	
F10.10	Alcohol abuse, uncomplicated
F10.120	Alcohol abuse with intoxication, uncomplicated
F10.121	Alcohol abuse with intoxication delirium
F10.129	Alcohol abuse with intoxication, unspecified
F10.14	Alcohol abuse with alcohol-induced mood disorder
F10.150	Alcohol abuse with alcohol-induced psychotic disorder with delusions
F10.151	Alcohol abuse with alcohol-induced psychotic disorder with hallucinations
F10.159	Alcohol abuse with alcohol-induced psychotic disorder, unspecified
F10.180	Alcohol abuse with alcohol-induced anxiety disorder
F10.181	Alcohol abuse with alcohol-induced sexual dysfunction
F10.182	Alcohol abuse with alcohol-induced sleep disorder
F10.188	Alcohol abuse with other alcohol-induced disorder
F10.19	Alcohol abuse with unspecified alcohol-induced disorder
F10.20	Alcohol dependence, uncomplicated
F10.21	Alcohol dependence, in remission
F10.220	Alcohol dependence with intoxication, uncomplicated
F10.221	Alcohol dependence with intoxication delirium
F10.229	Alcohol dependence with intoxication, unspecified
F10.230	Alcohol dependence with withdrawal, uncomplicated
F10.231	Alcohol dependence with withdrawal delirium
F10.232	Alcohol dependence with withdrawal with perceptual disturbance
F10.239	Alcohol dependence with withdrawal, unspecified
F10.24	Alcohol dependence with alcohol-induced mood disorder
F10.250	Alcohol dependence with alcohol-induced psychotic disorder with delusions
F10.251	Alcohol dependence with alcohol-induced psychotic disorder with hallucinations
F10.259	Alcohol dependence with alcohol-induced psychotic disorder, unspecified

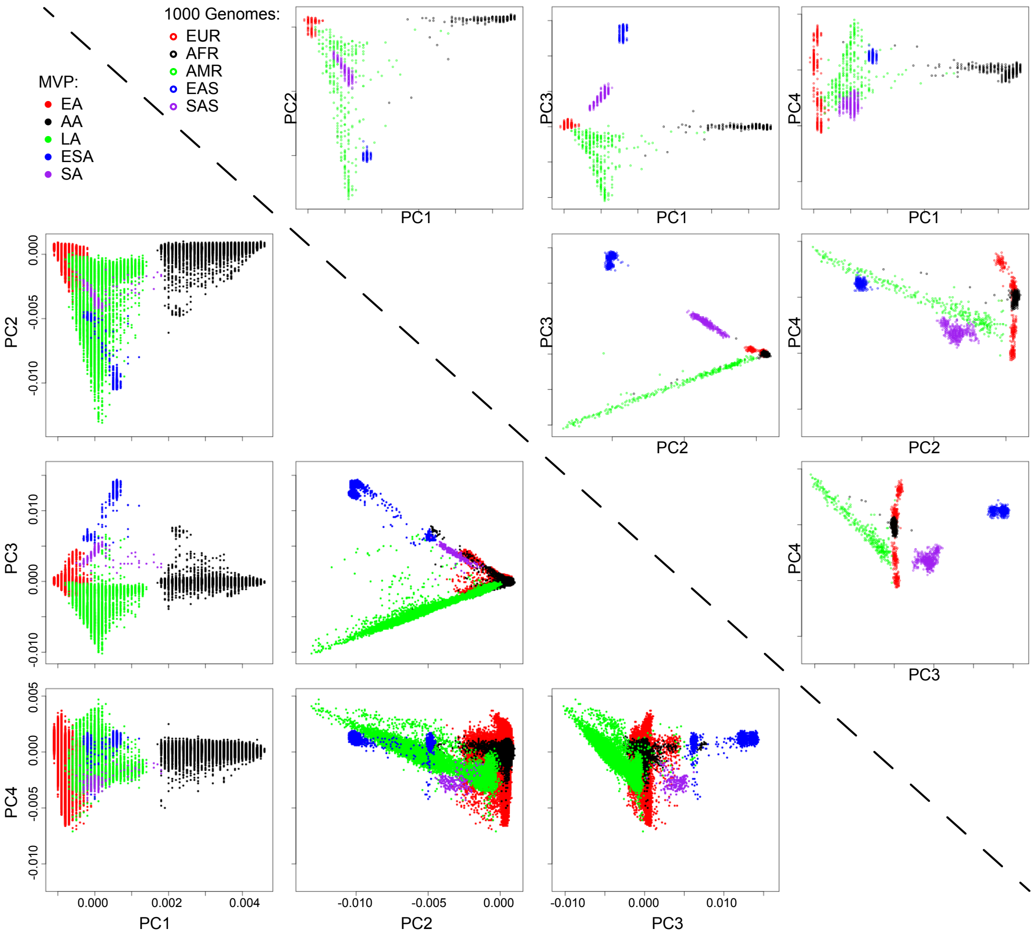

Supplementary Figure 1. PCA plot of MVP subjects and 1000 Genomes reference populations. PCA was performed using combined genetic data from MVP and 1000 Genomes, the first 10 PCs were used to cluster the MVP subjects into reference populations. Upper right panel, pairwise PC plots for 1000 Genomes reference populations, the first 4 PCs were presented. Lower left panel, pairwise PC plots for MVP sample with outliers removed, the first 4 PCs were presented. EUR, Europeans; AFR, Africans; AMR, admixed Americans; EAS, East Asians; SAS, South Asians.

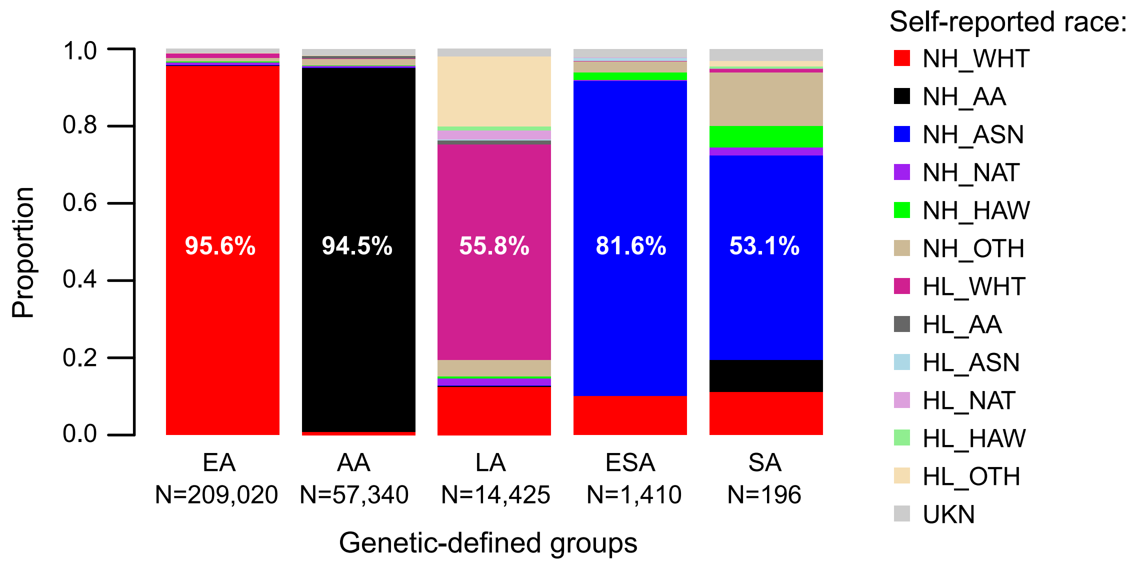

Supplementary Figure 2. Comparison of self-reported groups with genetically-defined groups. NH, non-Hispanic; HL, Hispanic or Latino; UKN, unknown; WHT, white; AA, Black or African American; ASN: Asian; NAT, American Indian or Alaska Native; HAW, Native Hawaiian or other Pacific; OTH, other or unknown.

a. EA

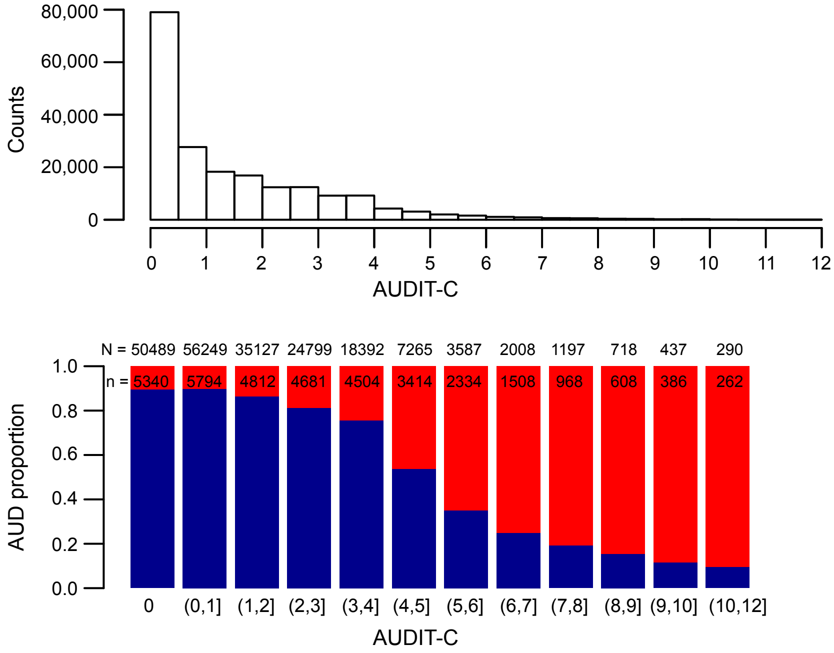

b. AA

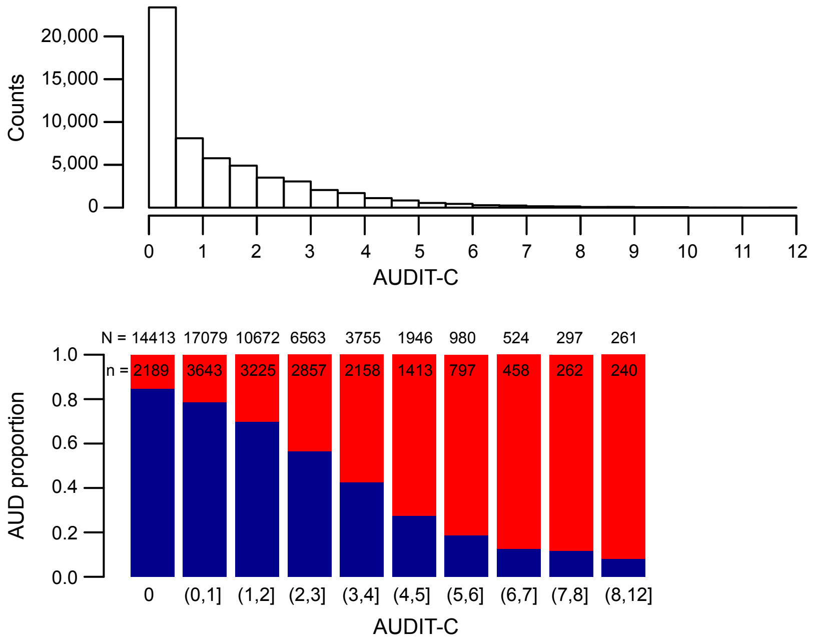

Supplementary Figure 3. Distribution of AUDIT-C and AUD by AUDIT-C score bins. Upper panel, the distribution of AUDIT-C scores; bottom panel, continuous AUDIT-C scores grouped into bins with sample size >100, presented as the proportion of AUD cases and controls in each bin. a), EA. b), AA. c), LA. d), ESA. e), SA.

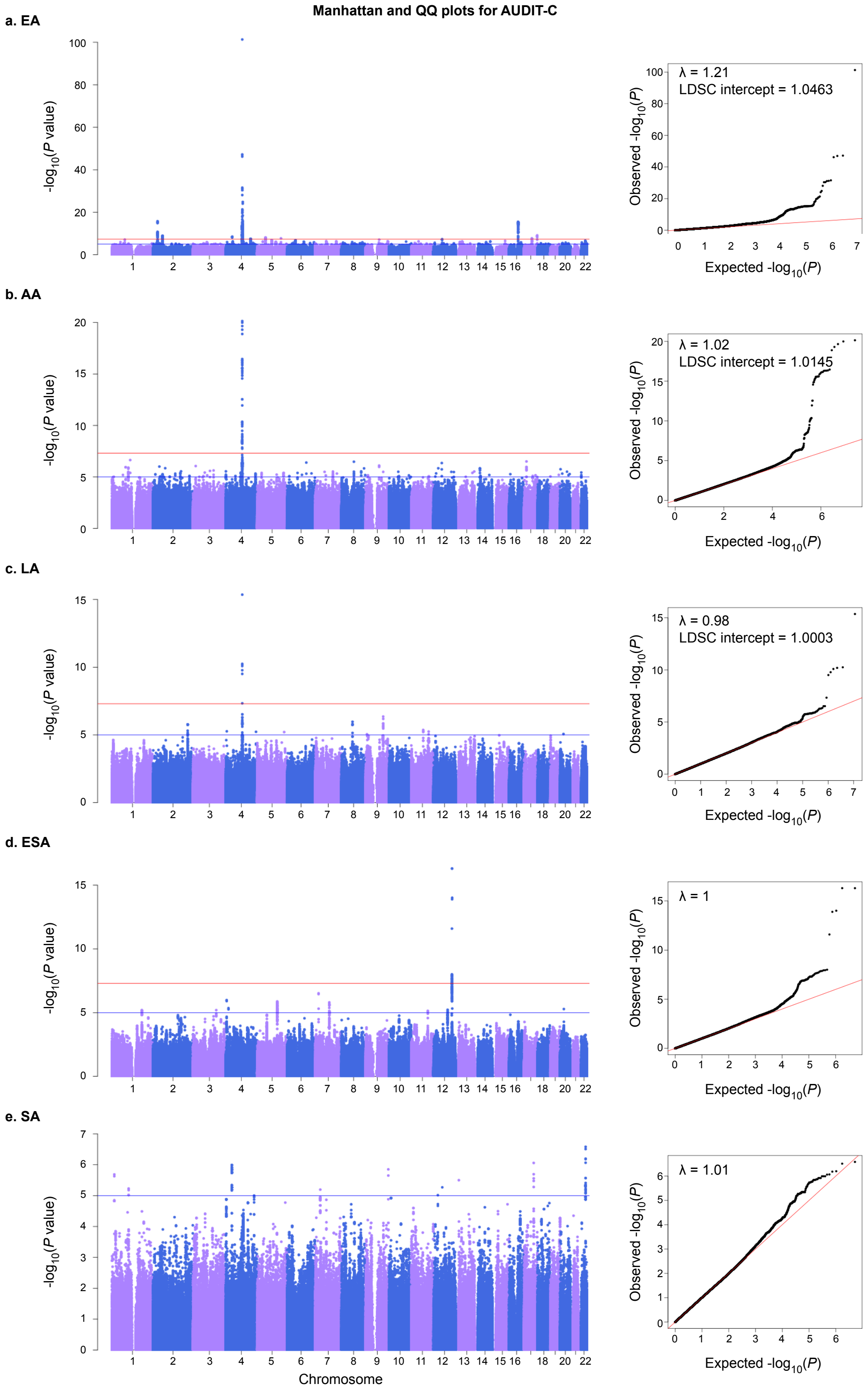

Supplementary Figure 4. Manhattan and QQ plots for AUDIT-C. LDSC intercept was estimated in populations with sample size ≥ 10,000. a), EA. b), AA. c), LA. d), ESA. e), SA.

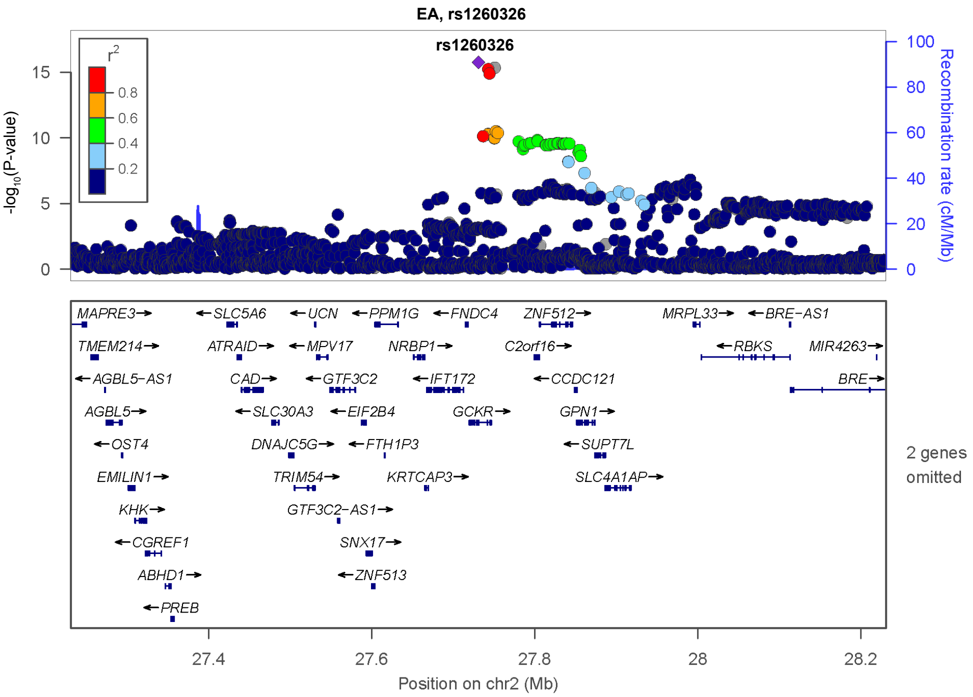

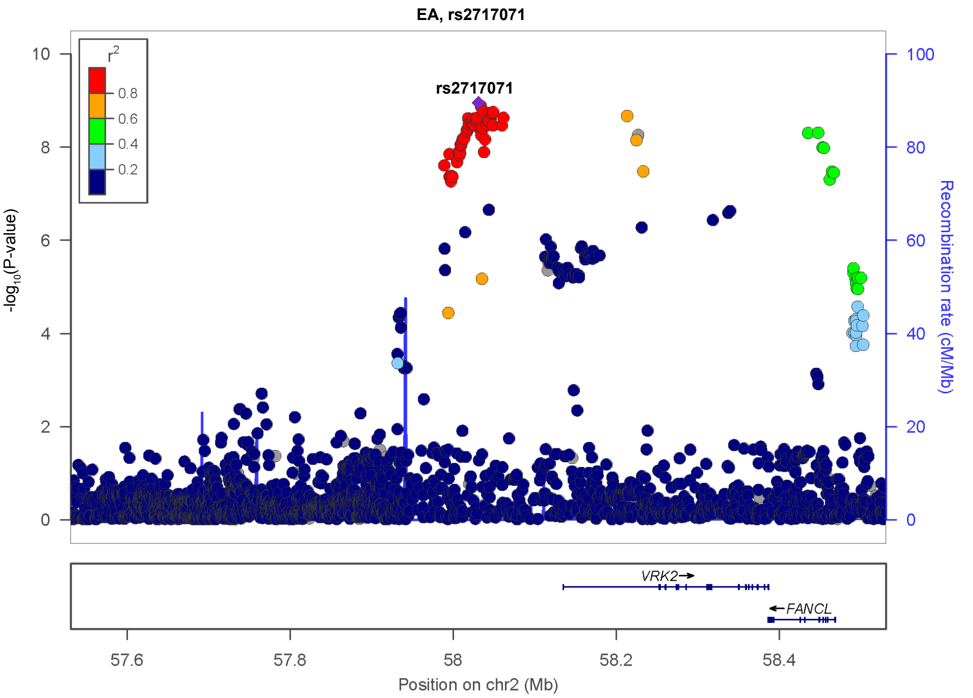

Supplementary Figure 5. Regional Manhattan plots for completely independent signals for AUDIT-C. Corresponding populations of 1000 Genomes were used as reference LD. No LD reference was used for the cross-population meta-analysis,.

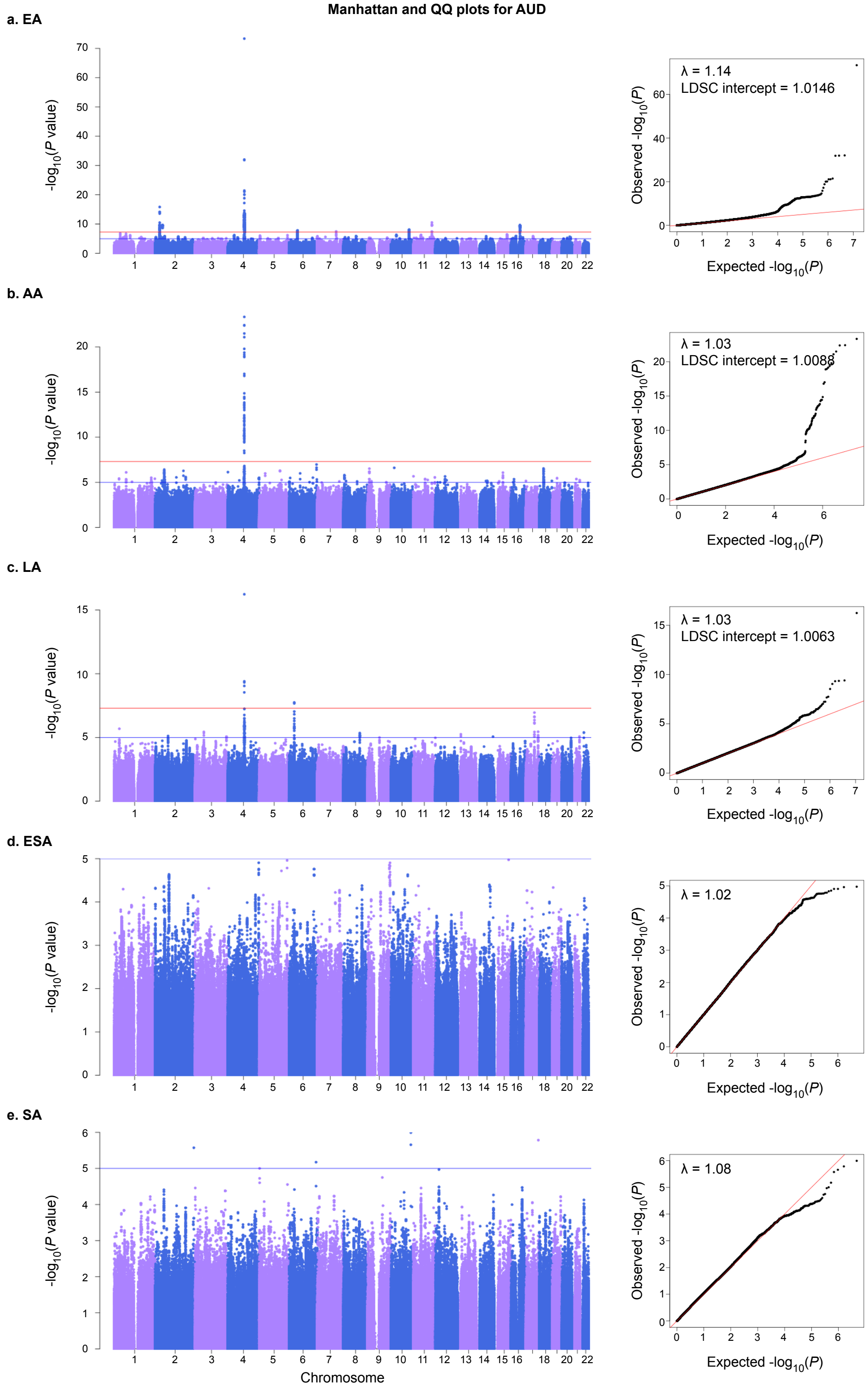

Supplementary Figure 6. Manhattan and QQ plots for AUD. LDSC intercept was estimated in populations with sample size ≥ 10,000. a), EA. b), AA. c), LA. d), ESA. e), SA.

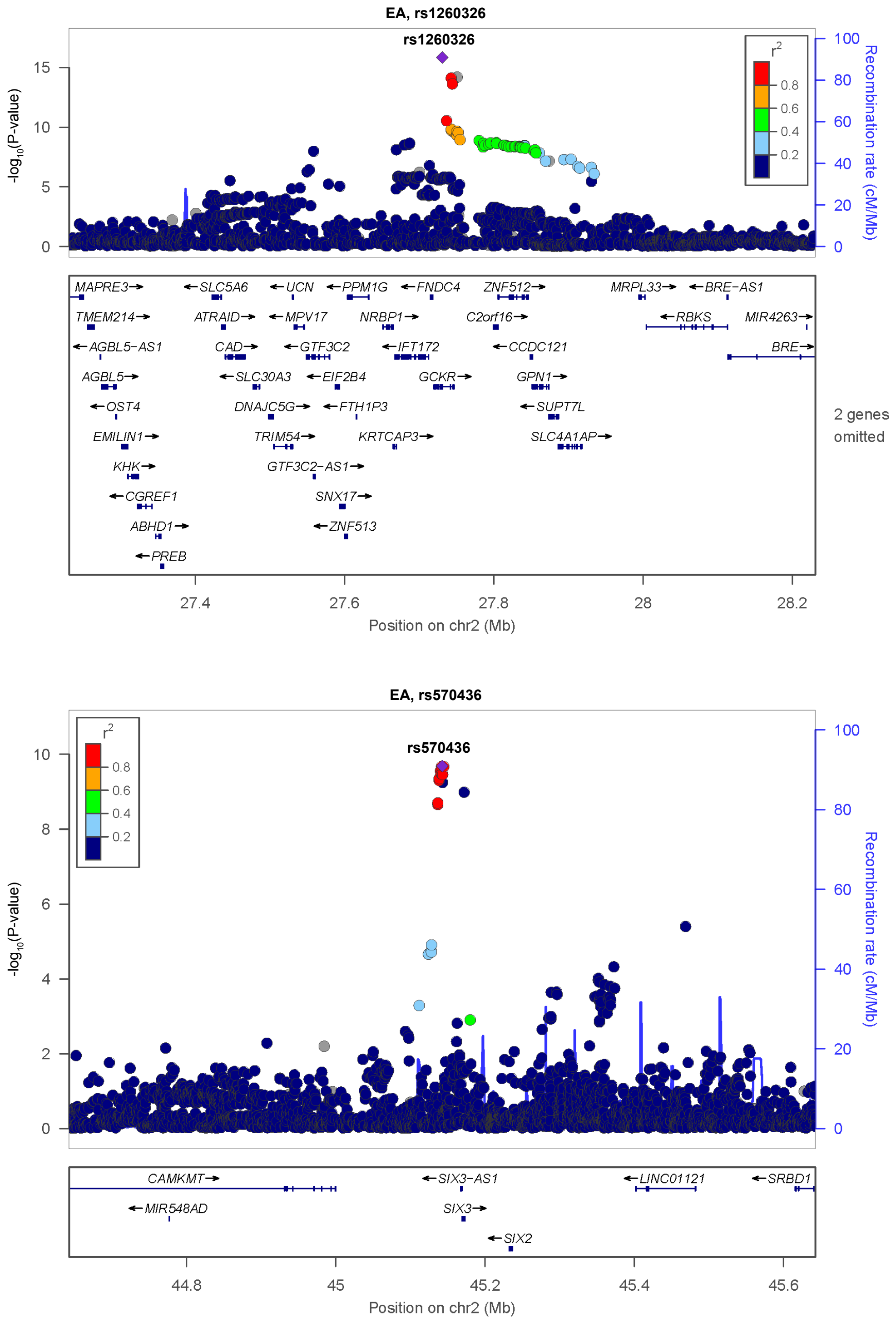

Supplementary Figure 7. Regional Manhattan plots for completely independent signals for AUD. Corresponding populations of 1000 Genomes were used as reference LD. For cross-populations meta-analysis, no LD reference was used.

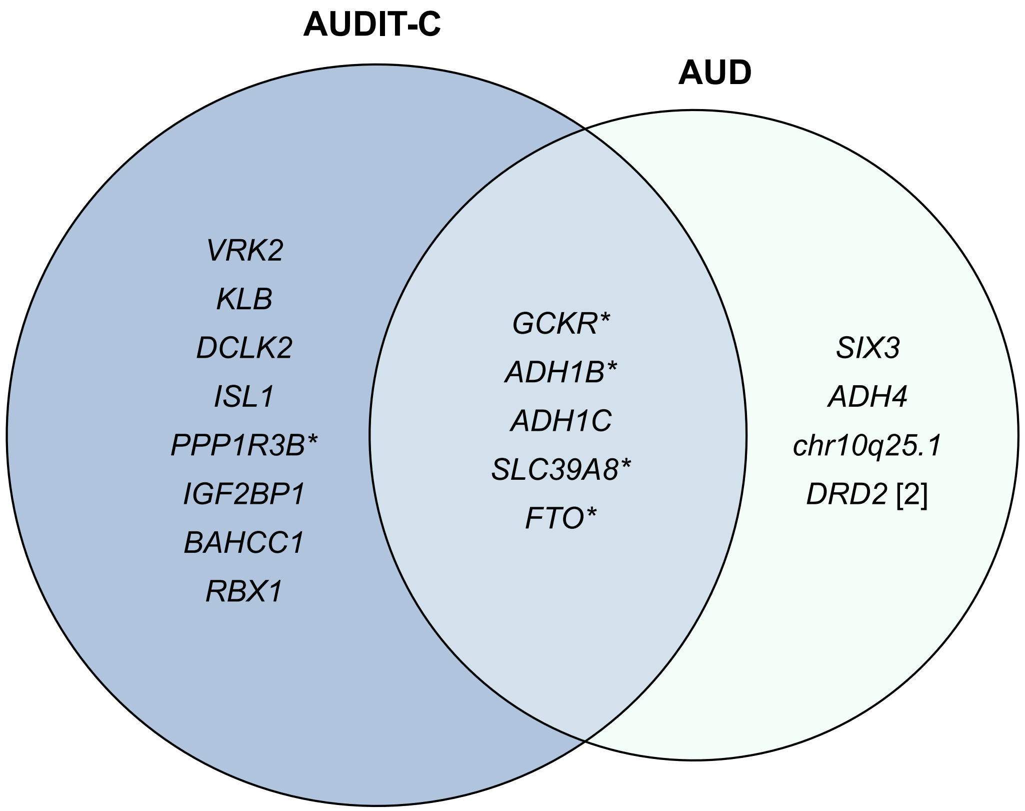

Supplementary Figure 8. Venn Diagram for overlap of genes containing variants associated with either AUDIT-C (left) or AUD (right) or both (middle) in the trans-population meta-analysis. Asterisk denotes pleiotropic genes (GWS associations found with other phenotypes, see supplemental table 34 for detail).

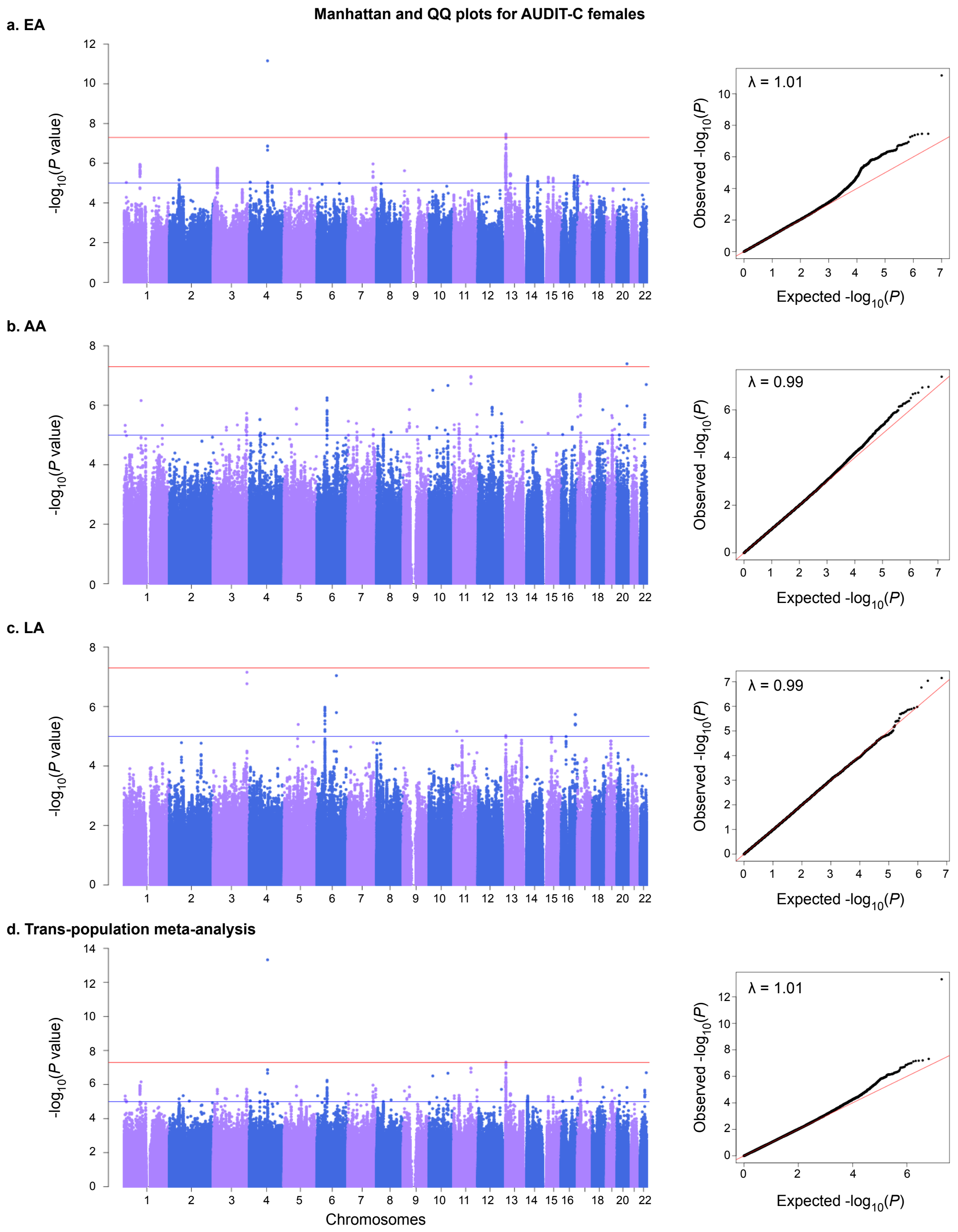

Supplementary Figure 9. Manhattan and QQ plots for AUDIT-C in females. a), EA. b), AA. c), LA. d), trans-population meta-analysis.

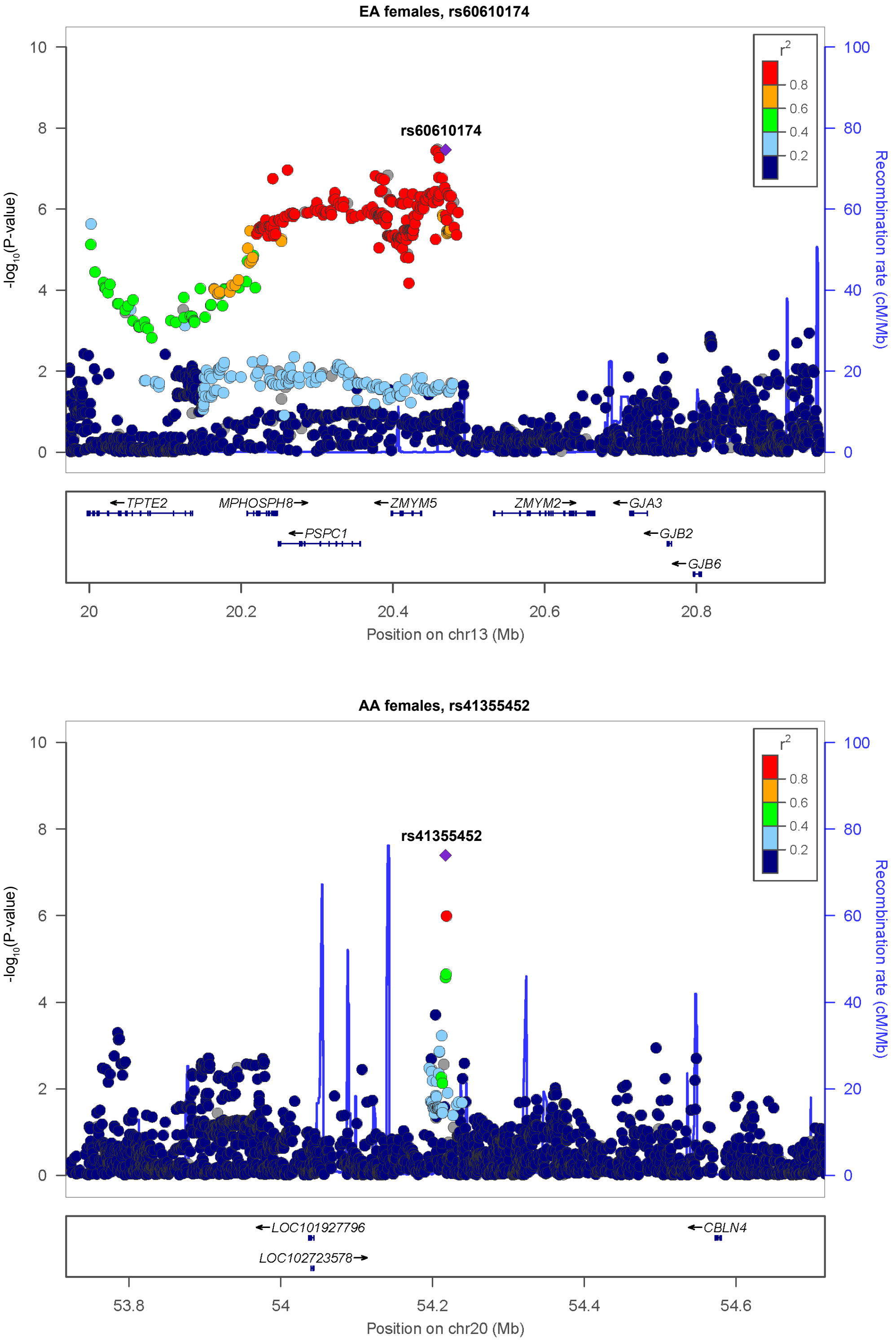

Supplementary Figure 10. Regional Manhattan plots for female-specific signals for AUDIT-C. Corresponding populations of 1000 Genomes were used as reference LD.

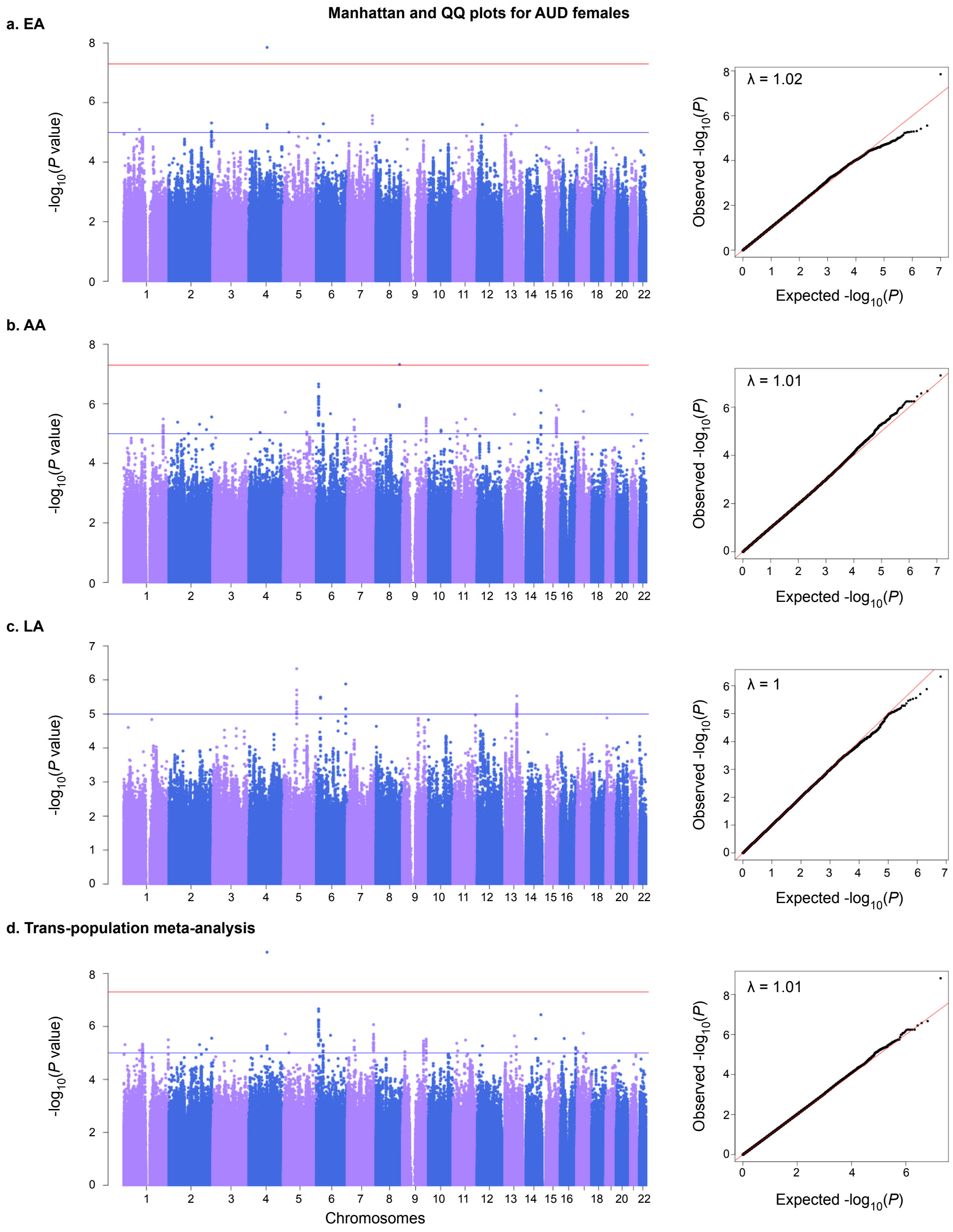

Supplementary Figure 11. Manhattan and QQ plots for AUD in females. a), EA. b), AA. c), LA. d), trans-population meta-analysis.

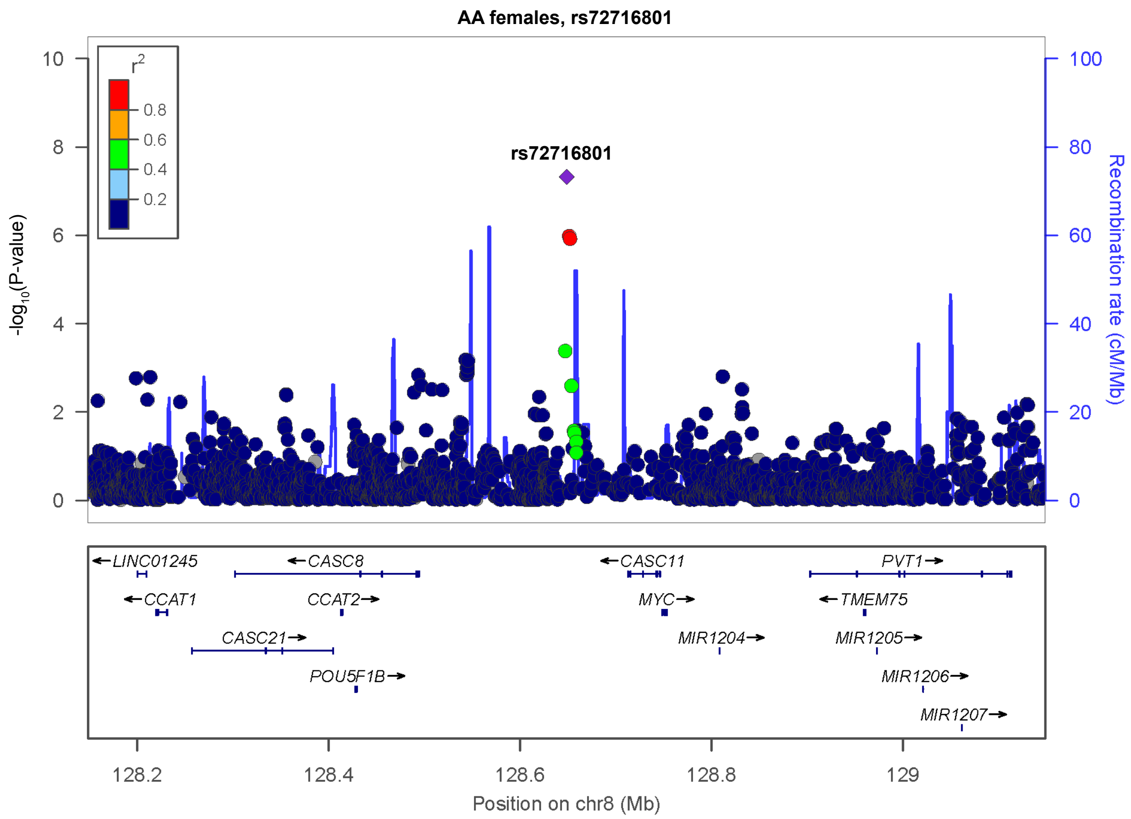

Supplementary Figure 12. Regional Manhattan plots for female-specific signal for AUD. AFR in 1000 Genomes was used as reference LD.

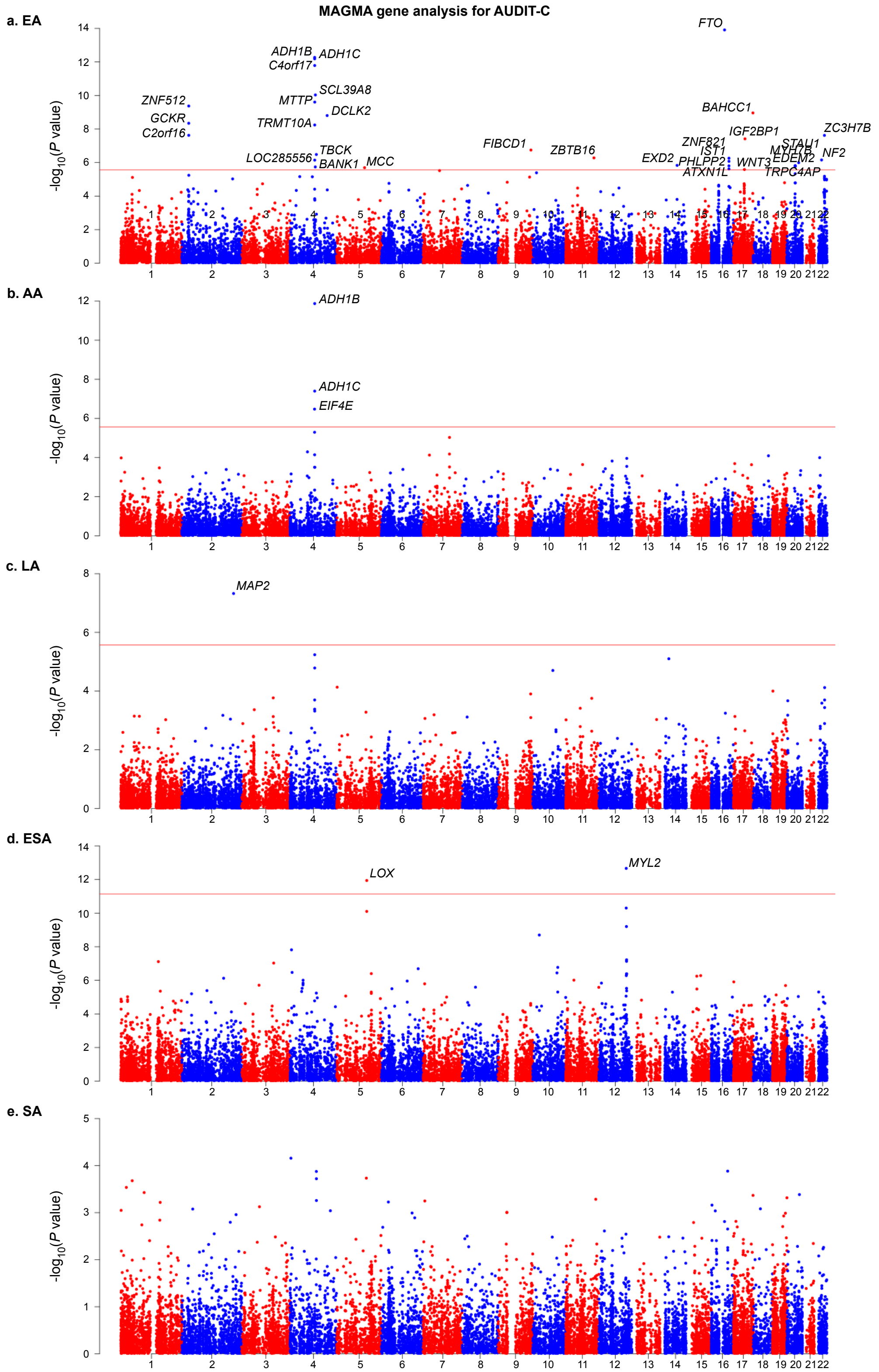

Supplementary Figure 13. Manhattan plots for gene-based association analysis for AUDIT-C. 18,575 autosome genes were analyzed and Bonferroni corrected genome-wide significant threshold is $P < 2.69 \times 10^{-6}$. a), EA. b), AA. c), LA. d), ESA. e), SA.

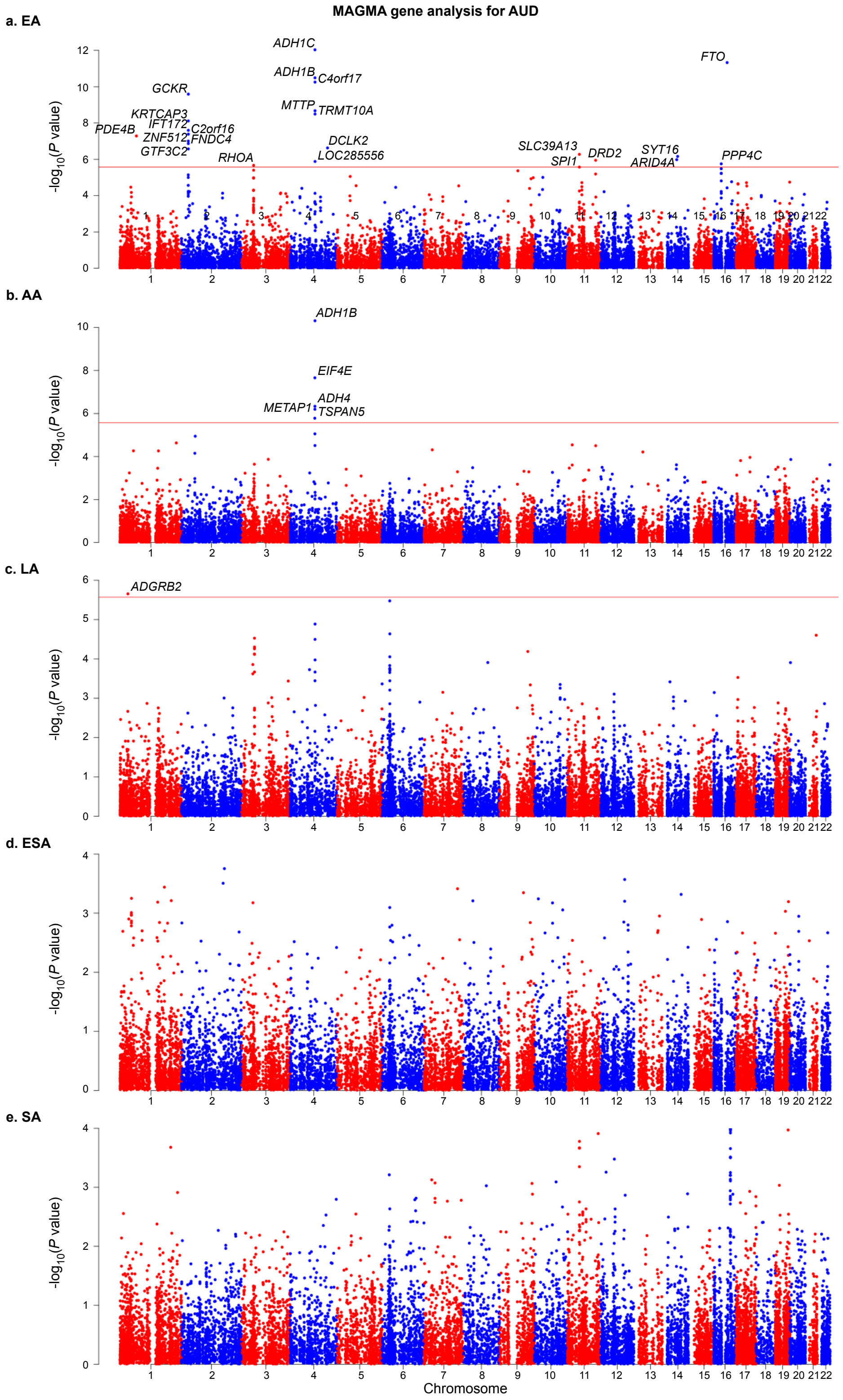

Supplementary Figure 14. Manhattan plots for gene-based association analysis for AUD. 18,575 autosome genes were analyzed and Bonferroni corrected genome-wide significant threshold is $P < 2.69 \times 10^{-6}$. a), EA. b), AA. c), LA. d), ESA. e), SA.

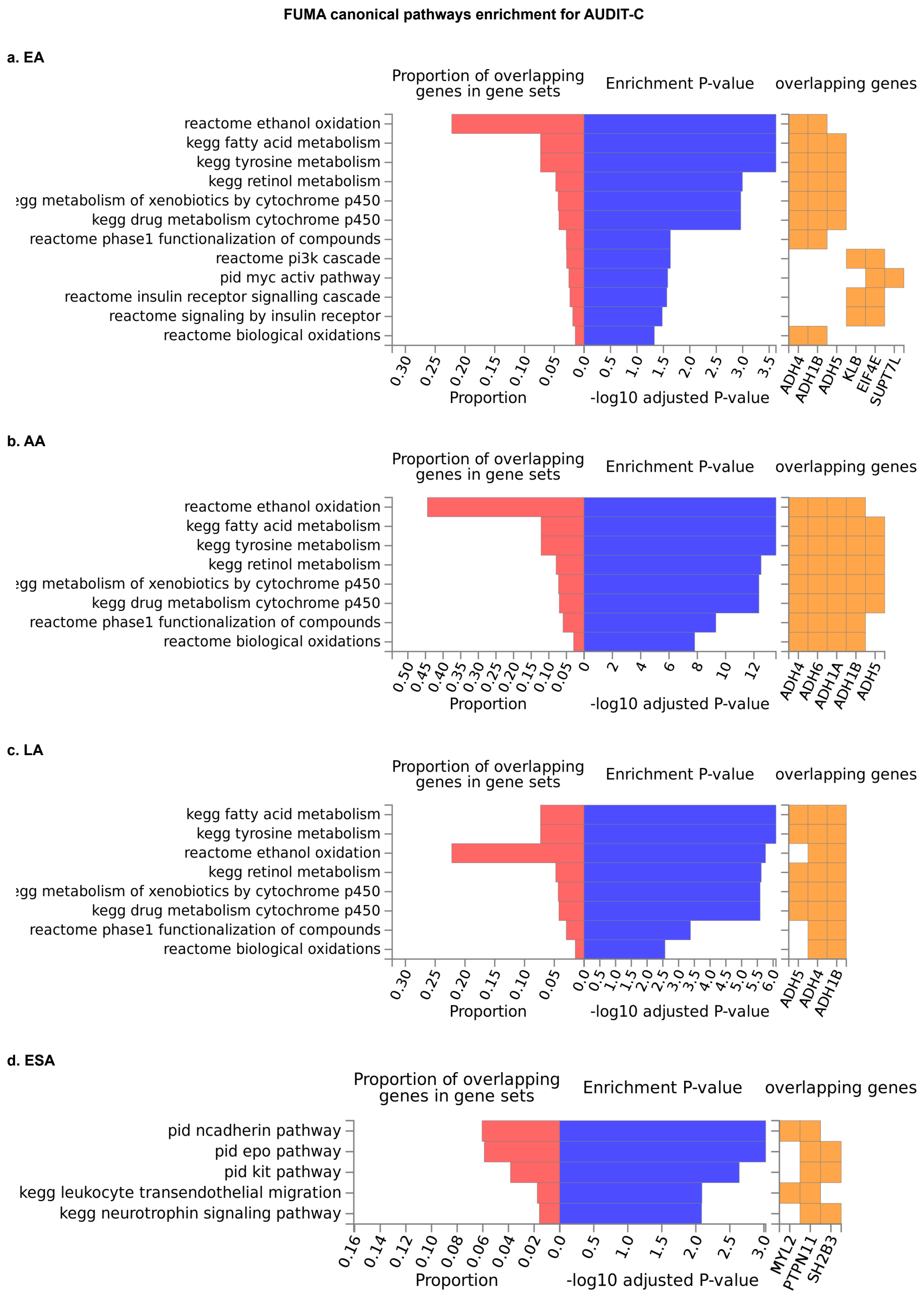

Supplementary Figure 15. FUMA canonical pathways enrichment for AUDIT-C. Only terms with adjusted p-value < 0.05 were presented. a), EA. b), AA. c), LA. d), ESA.

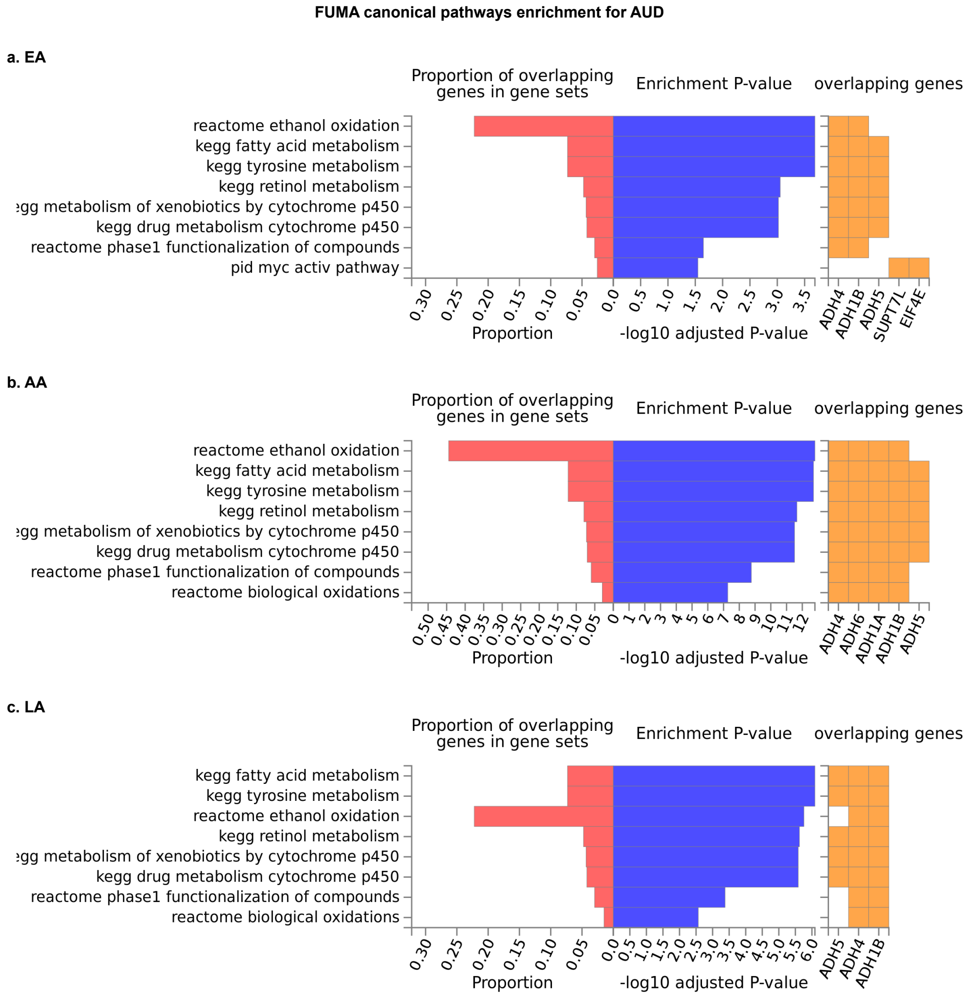

Supplementary Figure 16. FUMA canonical pathways enrichment for AUD. Only terms with adjusted p-value < 0.05 were presented. a), EA. b), AA. c), LA.

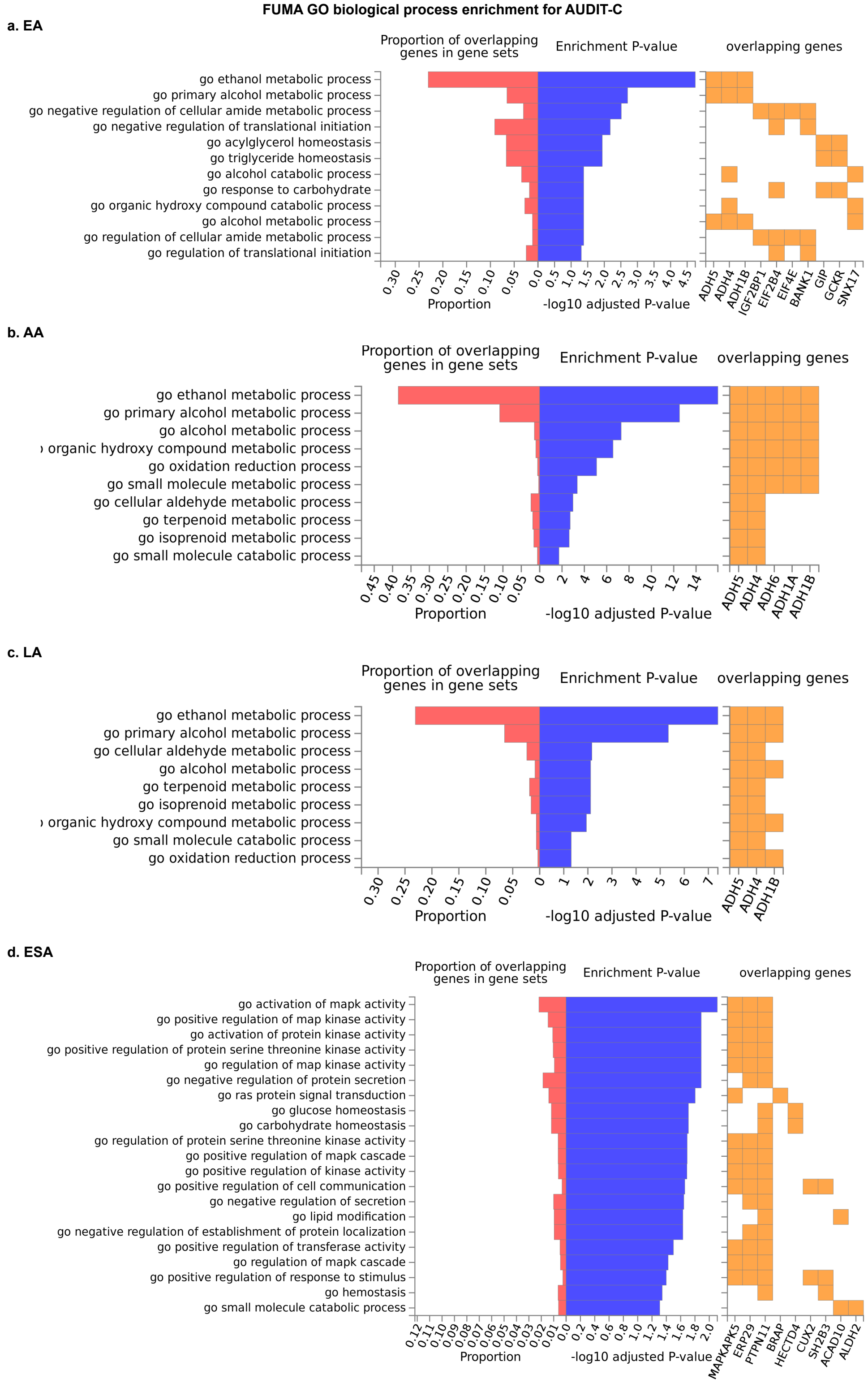

Supplementary Figure 17. FUMA GO biological process enrichment for AUDIT-C. Only terms with adjusted p-value < 0.05 were presented. a), EA. b), AA. c), LA. d), ESA.

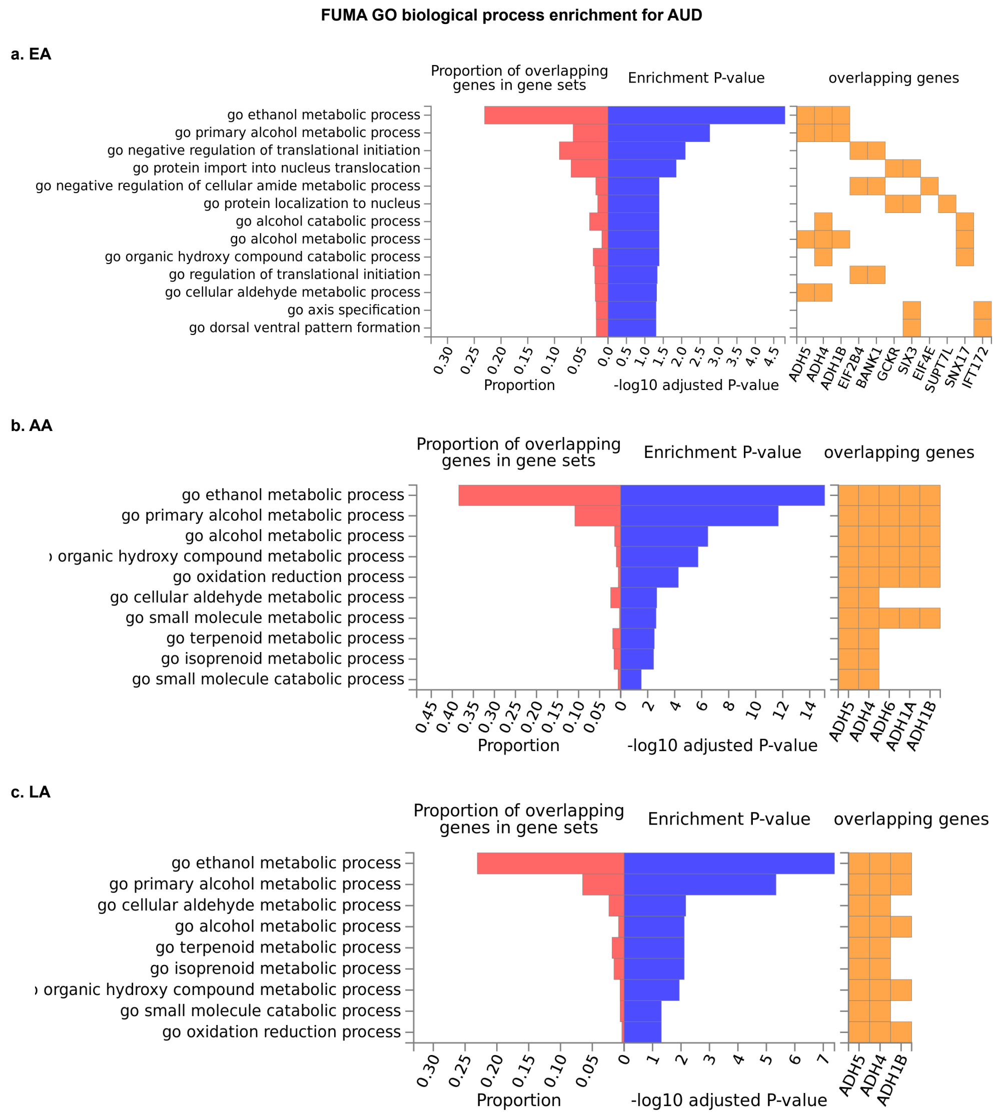

Supplementary Figure 18. FUMA GO biological process enrichment for AUD. Only terms with adjusted p-value < 0.05 were presented. a), EA. b), AA. c), LA.

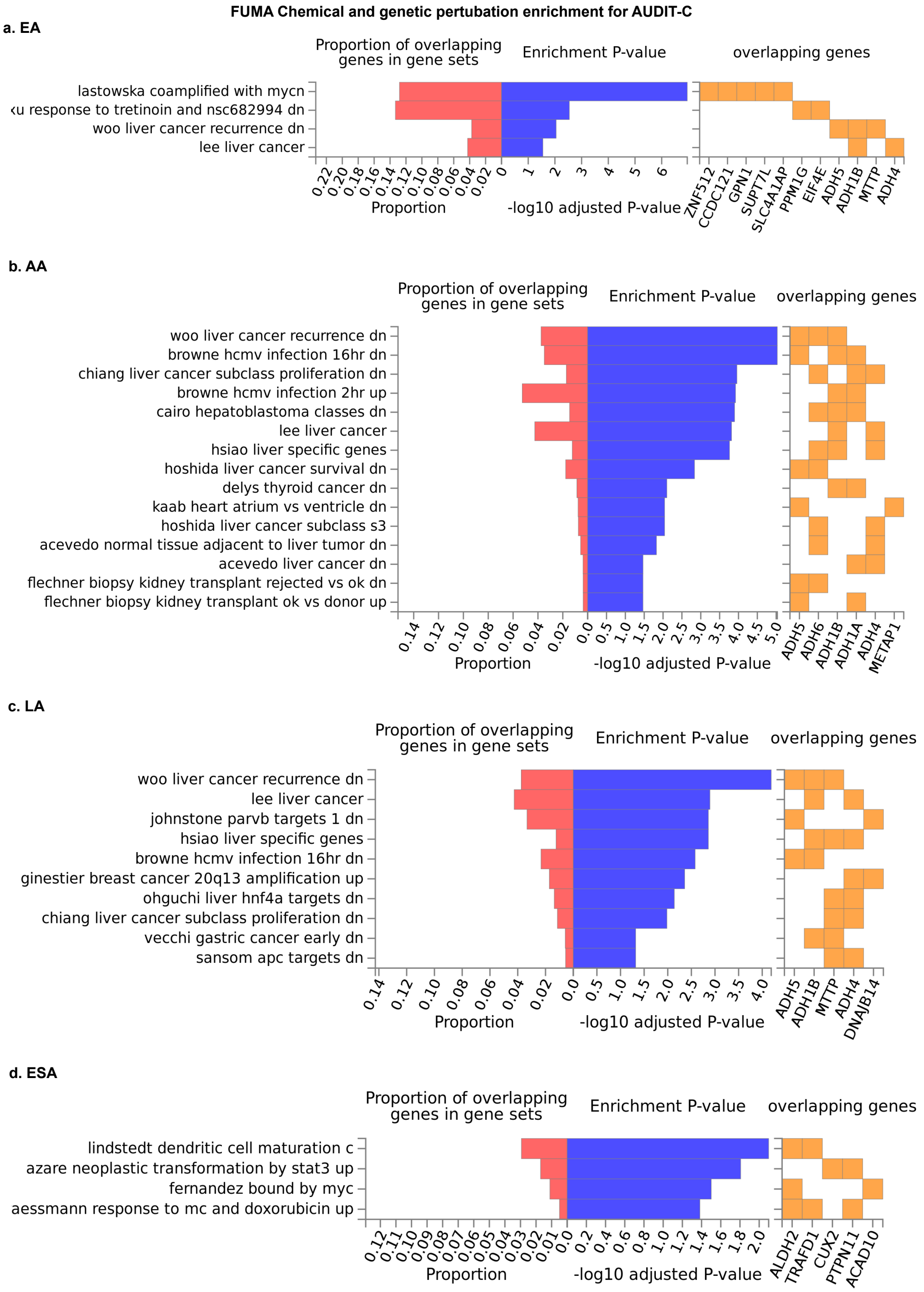

Supplementary Figure 19. FUMA Chemical and genetic pertubation enrichment for AUDIT-C. Only terms with adjusted p-value < 0.05 were presented. a), EA. b), AA. c), LA. d), ESA.

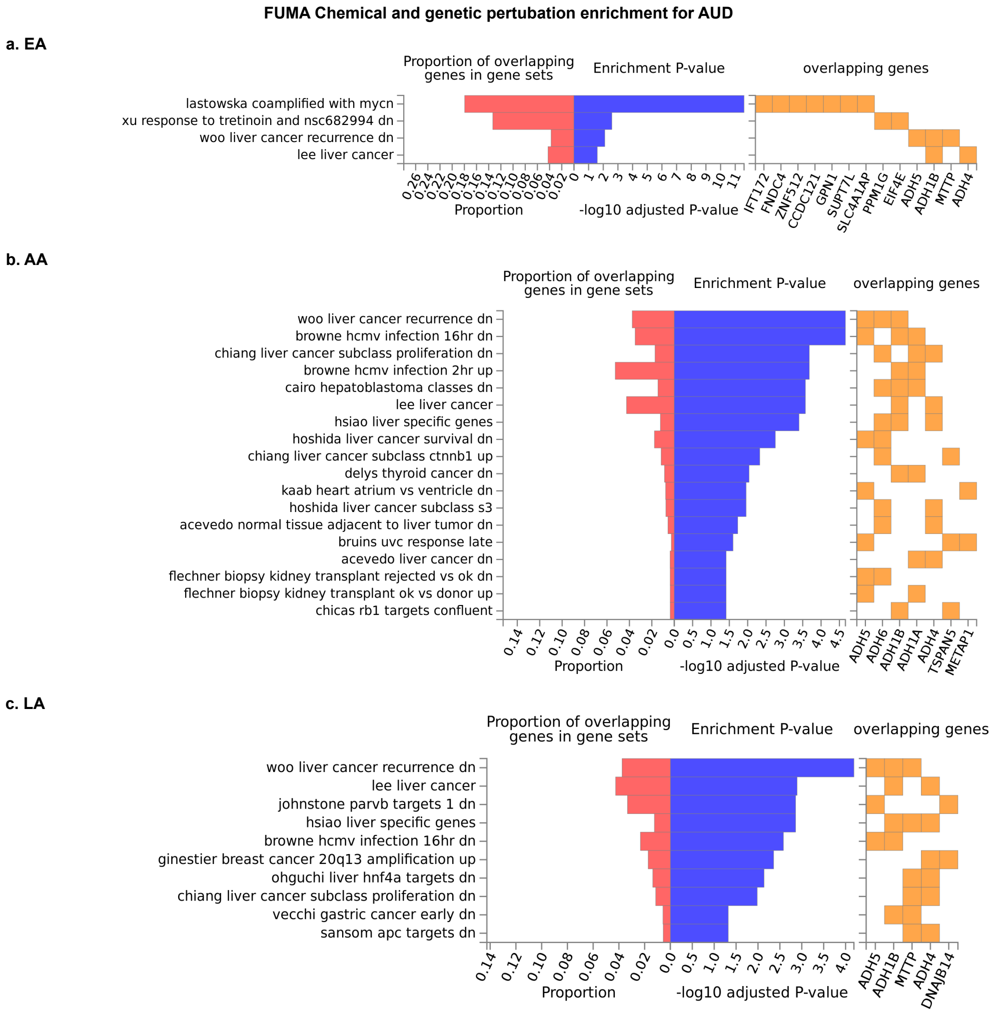

Supplementary Figure 20. FUMA Chemical and genetic perturbation enrichment for AUD. Only terms with adjusted p-value < 0.05 were presented. a), EA. b), AA. c), LA.

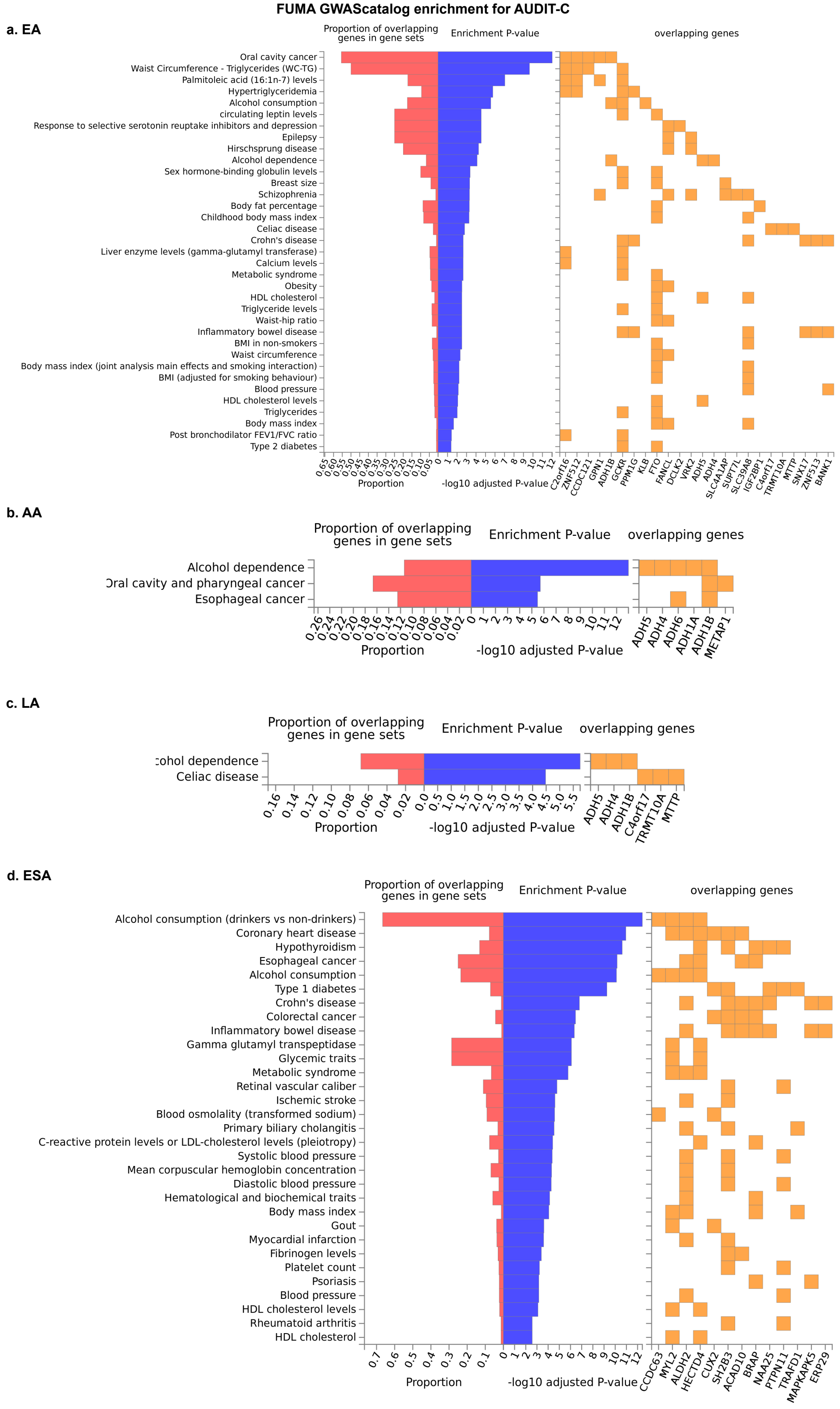

Supplementary Figure 21. FUMA GWAS catalog enrichment for AUDIT-C. Only terms with adjusted p-value < 0.05 were presented. a), EA. b), AA. c), LA. d), ESA.

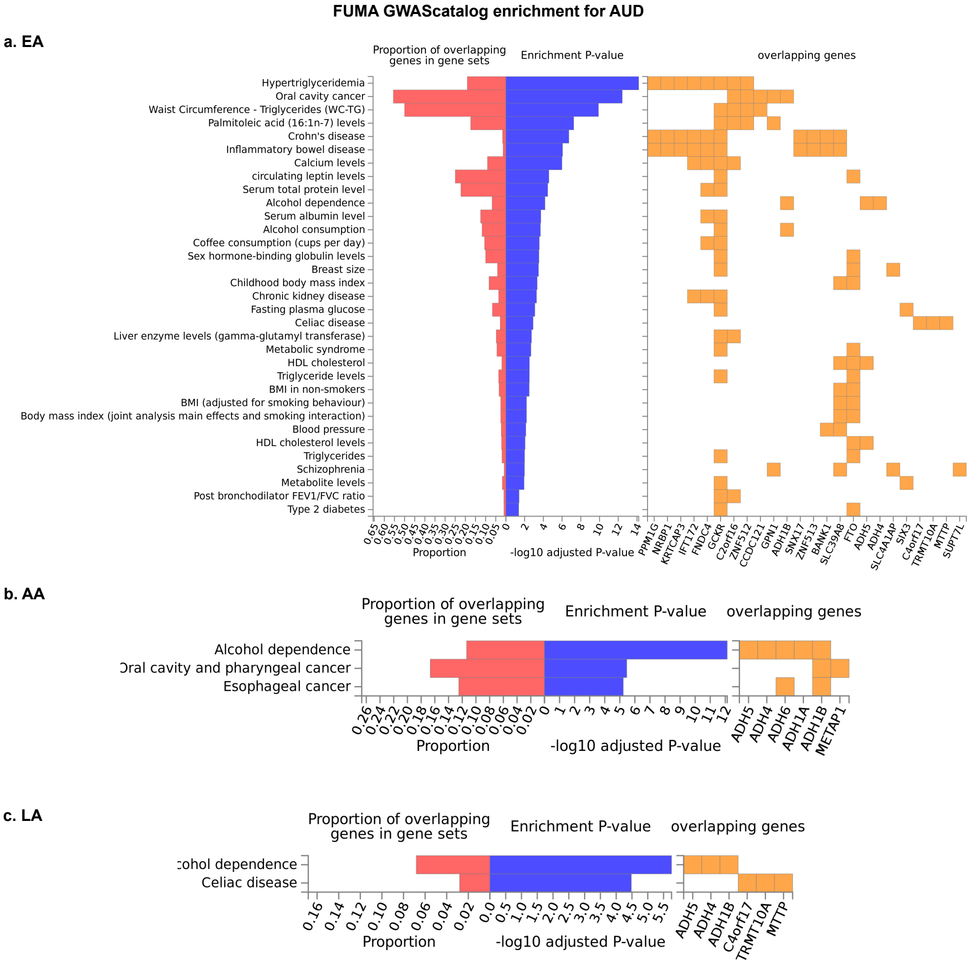

Supplementary Figure 22. FUMA GWAS catalog enrichment for AUD. Only terms with adjusted p-value < 0.05 were presented. a), EA. b), AA. c), LA.

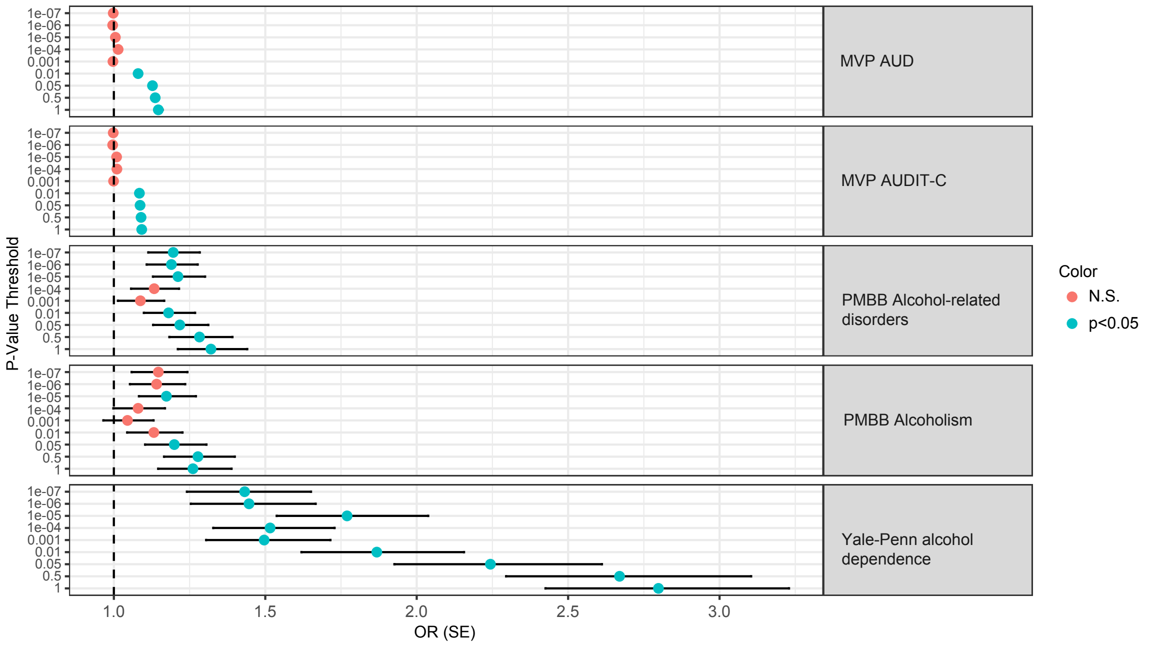

Supplementary Figure 23. EA AUDIT-C PRS. Association of AUDIT-C PRS generated for nine p-value thresholds in EA individuals with alcohol-related phenotypes in three datasets. OR: Odds Ratio, SE: Standard Error. Significant associations at p-value<0.05 are represented with a blue point.

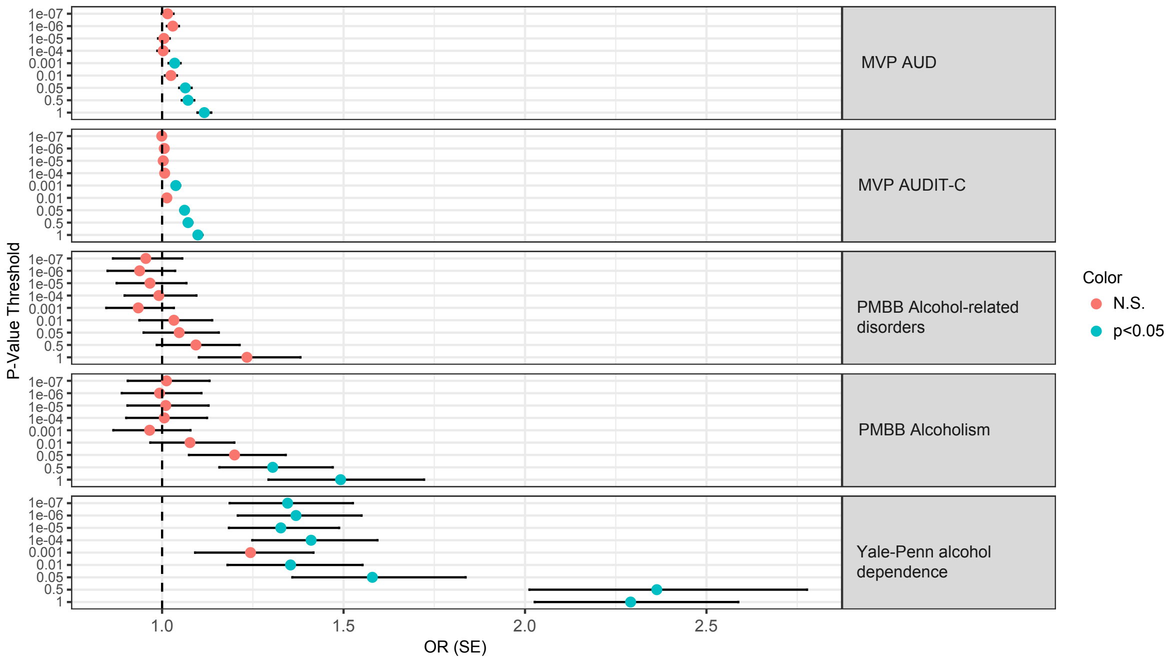

Supplementary Figure 24. AA AUDIT-C PRS. Association of AUDIT-C PRS generated for nine p-value thresholds in AA individuals with alcohol-related phenotypes in three datasets. OR: Odds Ratio, SE: Standard Error. Significant associations at p-value<0.05 are represented with a blue point.

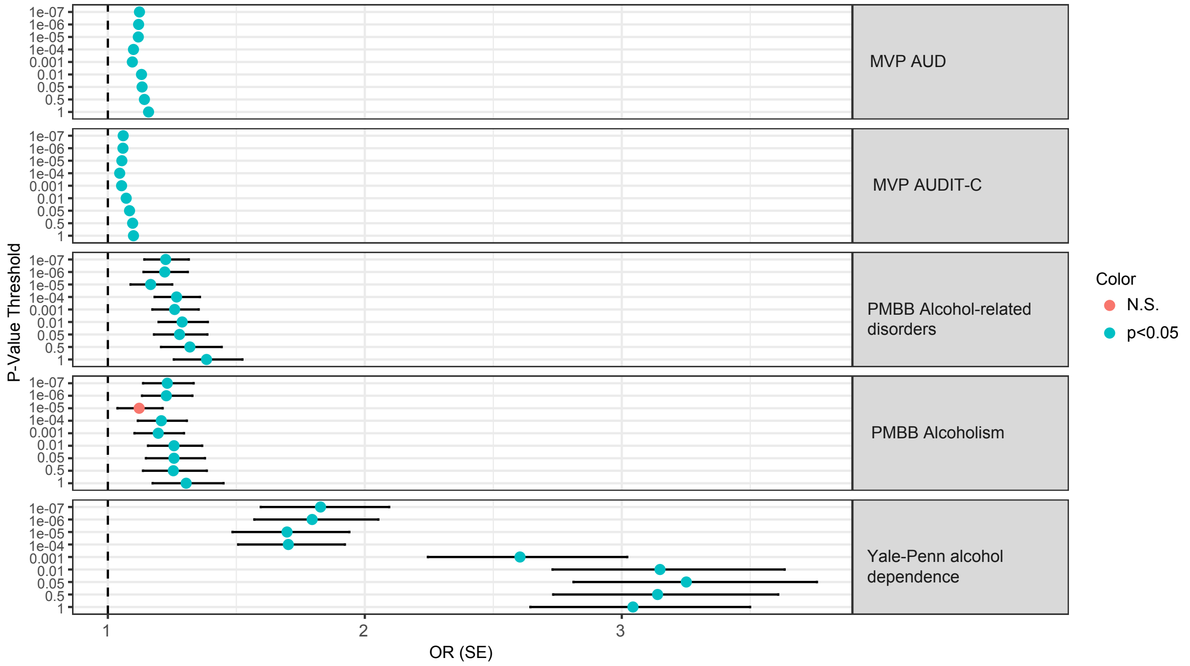

Supplementary Figure 25. EA AUD PRS. Association of AUD PRS generated for nine p-value thresholds in EA individuals with alcohol-related phenotypes in three datasets. OR: Odds Ratio, SE: Standard Error. Significant associations at p-value<0.05 are represented with a blue point.

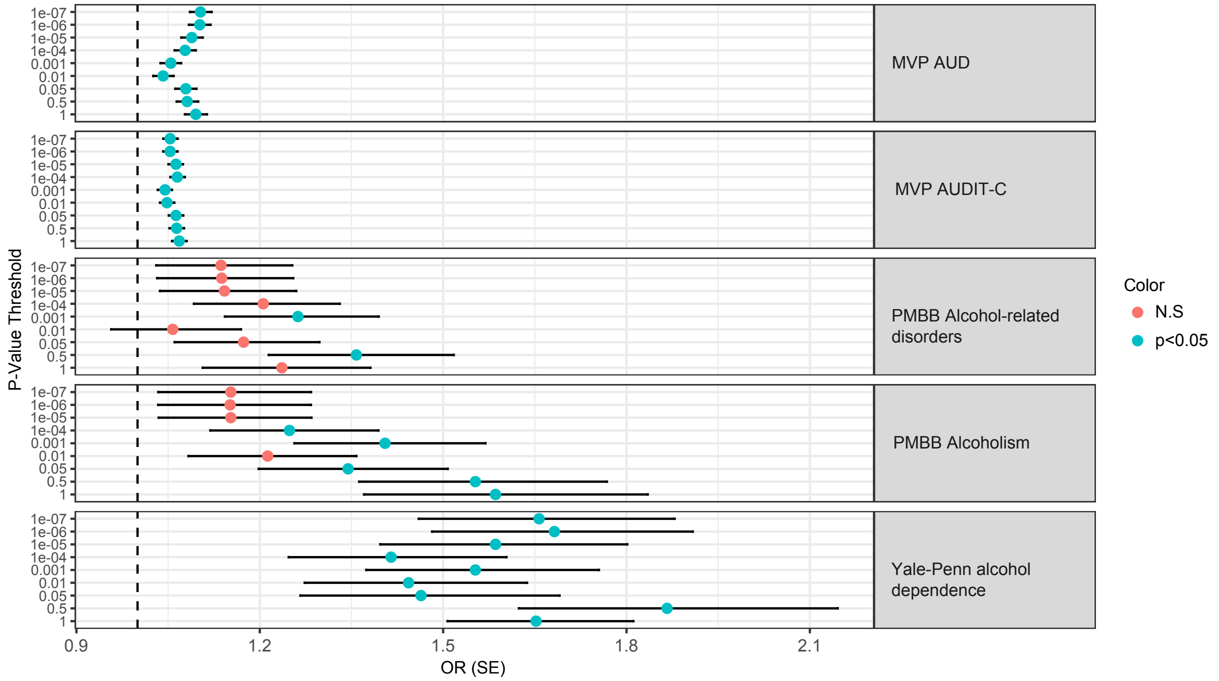

Supplementary Figure 26. AA AUD PRS. Association of AUD PRS generated for nine p-value thresholds in AA individuals with alcohol-related phenotypes in three datasets. OR: Odds Ratio, SE: Standard Error. Significant associations at p-value<0.05 are represented with a blue point.

Supplementary Note 1.

Million Veteran Program Consortium

MVP Executive Committee

- Co-Chair: J. Michael Gaziano, M.D., M.P.H.
- Co-Chair: Rachel Ramoni, D.M.D., Sc.D.
- Jim Breeling, M.D. (ex-officio)
- Kyong-Mi Chang, M.D.
- Grant Huang, Ph.D.
- Sumitra Muralidhar, Ph.D.
- Christopher J. O'Donnell, M.D., M.P.H.
- Philip S. Tsao, Ph.D.

MVP Program Office

- Sumitra Muralidhar, Ph.D.
- Jennifer Moser, Ph.D.

MVP Recruitment/Enrollment

- Recruitment/Enrollment Director/Deputy Director, Boston – Stacey B. Whitbourne, Ph.D.; Jessica V. Brewer, M.P.H.
- MVP Coordinating Centers
    - Clinical Epidemiology Research Center (CERC), West Haven – John Concato, M.D., M.P.H.
    - Cooperative Studies Program Clinical Research Pharmacy Coordinating Center, Albuquerque - Stuart Warren, J.D., Pharm D.; Dean P. Argyres, M.S.
    - Genomics Coordinating Center, Palo Alto – Philip S. Tsao, Ph.D.
    - Massachusetts Veterans Epidemiology Research Information Center (MAVERIC), Boston - J. Michael Gaziano, M.D., M.P.H.
    - MVP Information Center, Canandaigua – Brady Stephens, M.S.
- Core Biorepository, Boston – Mary T. Brophy M.D., M.P.H.; Donald E. Humphries, Ph.D.
- MVP Informatics, Boston – Nhan Do, M.D.; Shahpoor Shayan
- Data Operations/Analytics, Boston – Xuan-Mai T. Nguyen, Ph.D.

MVP Science

- Genomics - Christopher J. O'Donnell, M.D., M.P.H.; Saiju Pyarajan Ph.D.; Philip S. Tsao, Ph.D.
- Phenomics - Kelly Cho, M.P.H, Ph.D.
- Data and Computational Sciences – Saiju Pyarajan, Ph.D.
- Statistical Genetics – Elizabeth Hauser, Ph.D.; Yan Sun, Ph.D.; Hongyu Zhao, Ph.D.

MVP Local Site Investigators

--- Atlanta VA Medical Center (Peter Wilson)
1670 Clairmont Rd, Decatur, GA 30033
--- Bay Pines VA Healthcare System (Rachel McArdle)
10,000 Bay Pines Blvd Bay Pines FL 33744
--- Birmingham VA Medical Center (Louis Dellitalia)
700 S. 19th Street Birmingham AL 35233
--- Cincinnati VA Medical Center (John Harley)
3200 Vine Street, Cincinnati, OH 45220
--- Clement J. Zablocki VA Medical Center (Jeffrey Whittle)
5000 West National Avenue, Milwaukee, WI 53295
--- Durham VA Medical Center (Jean Beckham)
508 Fulton Street Durham, NC 27705
--- Edith Nourse Rogers Memorial Veterans Hospital (John Wells)
200 Springs Road, Bedford, MA 01730
--- Edward Hines, Jr. VA Medical Center (Salvador Gutierrez)
5000 South 5th Avenue, Hines, IL 60141
--- Fayetteville VA Medical Center (Gretchen Gibson)
1100 N College Ave, Fayetteville, AR 72703
--- VA Health Care Upstate New York (Laurence Kaminsky)
113 Holland Avenue Albany NY 12208
--- New Mexico VA Health Care System (Gerardo Villareal)
1501 San Pedro Drive, S.E.Albuquerque, NM 87108
--- VA Boston Healthcare System (Scott Kinlay)
150 S. Huntington Avenue, Boston, MA 02130
--- VA Western New York Healthcare System (Junzhe Xu)
3495 Bailey Avenue Buffalo, NY 14215-1199
--- Ralph H. Johnson VA Medical Center (Mark Hamner)
109 Bee Street, Mental Health Research, Charleston, SC 29401
--- Wm. Jennings Bryan Dorn VA Medical Center (Kathlyn Sue Haddock) 6439
Garners Ferry Road, Columbia, SC 29209
--- VA North Texas Health Care System (Sujata Bhushan)
4500 S. LANCASTER ROAD, DALLAS, TX 75216
--- Hampton VA Medical Center (Pran Iruvanti) 100
Emancipation Drive, Hampton, VA 23667
--- Hunter Holmes McGuire VA Medical Center (Michael Godschalk)
1201 Broad Rock Blvd., Richmond, VA 23249
--- Iowa City VA Health Care System (Zuhair Ballas)
601 Highway 6 West, Iowa City, IA 52246-2208
--- Jack C. Montgomery VA Medical Center (Malcolm Buford)
1011 Honor Heights Dr., Muskogee, OK 74401
--- James A. Haley Veterans' Hospital (Stephen Mastorides)
13000 Bruce B. Downs Blvd., Tampa, FL 33612
--- Louisville VA Medical Center (Jon Klein) 800
Zorn Avenue, Louisville, KY 40206
--- Manchester VA Medical Center (Nora Ratcliffe)
718 Smyth Road, Manchester, NH 03104

--- Miami VA Health Care System (Hermes Florez)
1201 NW 16th Street, 11 GRC, Miami FL 33125
--- Michael E. DeBakey VA Medical Center (Alan Swann)
2002 Holcombe Blvd. Houston TX 77030
--- Minneapolis VA Health Care System (Maureen Murdoch)
One Veterans Drive Minneapolis MN 55417
--- N. FL/S. GA Veterans Health System (Peruvemba Sriram)
1601 SW Archer Road, Gainesville, FL 32608
--- Northport VA Medical Center (Shing Shing Yeh) 79
Middleville Road, Northport, NY 11768
--- Overton Brooks VA Medical Center (Ronald Washburn)
510 East Stoner Ave, Shreveport, LA 71101
--- Philadelphia VA Medical Center (Darshana Jhala)
3900 Woodland Avenue, Philadelphia, PA 19104
--- Phoenix VA Health Care System (Samuel Aguayo)
650 E. Indian School Road, Phoenix, AZ 85012
--- Portland VA Medical Center (David Cohen)
3710 SW U.S. Veterans Hospital Road, Portland, OR 97239
--- Providence VA Medical Center (Satish Sharma)
830 Chalkstone Avenue, Providence, RI 02908
--- Richard Roudebush VA Medical Center (John Callaghan)
1481 West 10th Street, Indianapolis, IN 46202
--- Salem VA Medical Center (Kris Ann Oursler) 1970
Roanoke Blvd.,Salem, VA 24153
--- San Francisco VA Health Care System (Mary Whooley)
4150 Clement Street, San Francisco, CA 94121
--- South Texas Veterans Health Care System (Sunil Ahuja)
7400 Merton Minter Boulevard, San Antonio, TX 78229
--- Southeast Louisiana Veterans Health Care System (Amparo Gutierrez)
2400 Canal Street, New Orleans, LA 70119
--- Southern Arizona VA Health Care System (Ronald Schifman)
3601 S 6th Ave, Tucson, AZ 85723
--- Sioux Falls VA Health Care System (Jennifer Greco)
2501 W 22nd St, Sioux Falls, SD 57105
--- St. Louis VA Health Care System (Michael Rauchman)
915 North Grand Blvd., St. Louis, MO 63106
--- Syracuse VA Medical Center (Richard Servatius)
800 Irving Avenue, Syracuse, NY 13210
--- VA Eastern Kansas Health Care System (Mary Oehlert)
4101 S 4th Street Trafficway, Leavenworth, KS 66048
--- VA Greater Los Angeles Health Care System (Agnes Wallbom)
11301 Wilshire Blvd Los Angeles, CA 90073
--- VA Loma Linda Healthcare System (Ronald Fernando)
11201 Benton Street, Loma Linda, CA 92357
--- VA Long Beach Healthcare System (Timothy Morgan)
5901 East 7th Street Long Beach CA 90822
--- VA Maine Healthcare System (Todd Stapley)
1 VA Center, Augusta, ME 04330

--- VA New York Harbor Healthcare System (Scott Sherman) 423 East 23rd Street New York, NY 10010
--- VA Pacific Islands Health Care System (Gwenevere Anderson) 459 Patterson Rd, Honolulu, HI 96819
--- VA Palo Alto Health Care System (Philip Tsao) 3801 Miranda Avenue Palo Alto, CA 94304-1290
--- VA Pittsburgh Health Care System (Elif Sonel) University Drive, Pittsburgh, PA 15240
--- VA Puget Sound Health Care System (Edward Boyko) 1660 S. Columbian Way Seattle, WA 98108-1597
--- VA Salt Lake City Health Care System (Laurence Meyer) 500 Foothill Drive Salt Lake City, UT 84148
--- VA San Diego Healthcare System (Samir Gupta) 3350 La Jolla Village Drive, San Diego, CA 92161
--- VA Southern Nevada Healthcare System (Joseph Fayad) 6900 North Pecos Road, North Las Vegas, NV 89086
--- VA Tennessee Valley Healthcare System (Adriana Hung) 1310 24th Ave. South Nashville, TN 37212
--- Washington DC VA Medical Center (Jack Lichy) 50 Irving St, Washington, D. C. 20422
--- W.G. (Bill) Hefner VA Medical Center (Robin Hurley) 1601 Brenner Ave, Salisbury, NC 28144
--- White River Junction VA Medical Center (Brooks Robey) 163 Veterans Drive, White River Junction, VT 05009
--- William S. Middleton Memorial Veterans Hospital (Robert Striker) 2500 Overlook Terrace, Madison, WI 53705
