# Supplementary material for: Genome-wide association study of alcohol consumption and use disorder in 274,424 individuals from multiple populations
Source: Nat Commun. 2019 Apr 2;10:1499. doi: 10.1038/s41467-019-09480-8 (PMC6445072; doi:10.1038/s41467-019-09480-8)
Supplement: Supplementary file 3 — Description of Additional Supplementary Files [file 41467_2019_9480_MOESM3_ESM.pdf]

Description of Supplementary Data

Supplementary Data 1: Genome-wide significant associations for AUDIT-C in each population.
Supplementary Data 2: Genome-wide significant associations for AUD in each population.
Supplementary Data 3: Female-specific genome-wide significant associations for AUDIT-C in each population.
Supplementary Data 4: Female-specific genome-wide significant associations for AUD in AA.
Supplementary Data 5: Conditional analyses of lead SNPs on chr4 for AUDIT-C.
Supplementary Data 6: Conditional analyses of lead SNPs on chr4 for AUD.
Supplementary Data 7: Genome-wide significant associations for AUDIT-C with AUD as a covariate.
Supplementary Data 8: Genome-wide significant associations for AUD with AUDIT-C as a covariate.
Supplementary Data 9: Secondary GWAS for AUDIT-C adjusting for BMI.
Supplementary Data 10: Secondary GWAS for AUD adjusting for BMI.
Supplementary Table 11: Canonical pathways for AUDIT-C GWAS.
Supplementary Data 12: Canonical pathways for AUD GWAS.
Supplementary Data 13: GO biological processes for AUDIT-C GWAS.
Supplementary Data 14: GO biological processes for AUD GWAS.
Supplementary Data 15: Chemical and genetic perturbation for AUDIT-C GWAS.
Supplementary Data 16: Chemical and genetic perturbation for AUD GWAS.
Supplementary Data 17: GWAS catalog enrichment for AUDIT-C GWAS.
Supplementary Data 18: GWAS catalog enrichment for AUD GWAS.
Supplementary Data 19: Cell line partitioning heritability enrichment for AUDIT-C GWAS using baseline model.
Supplementary Data 20: Cell line partitioning heritability enrichment for AUD GWAS using baseline model.
Supplementary Data 21: Cell type group partitioning heritability enrichment for AUDIT-C GWAS.
Supplementary Data 22: Cell type group partitioning heritability enrichment for AUD GWAS.
Supplementary Data 23: Mouse brain cell type partitioning heritability enrichment for AUDIT-C GWAS using Cahoy gene expression data.
Supplementary Table 24: Mouse brain cell type partitioning heritability enrichment for AUD GWAS using Cahoy gene expression data.
Supplementary Data 25: Tissue or cell type partitioning heritability enrichment for AUDIT-C GWAS using Franke-lab gene expression data.
Supplementary Data 26: Tissue or cell type partitioning heritability enrichment for AUD GWAS using Franke-lab gene expression data.
Supplementary Data 27: Tissue or cell type partitioning heritability enrichment for AUDIT-C GWAS using GTEx gene expression data.
Supplementary Data 28: Tissue or cell type partitioning heritability enrichment for AUD GWAS using GTEx gene expression data.
Supplementary Data 29: Mouse immune cell type partitioning heritability enrichment for AUDIT-C GWAS using ImmGen gene expression data.
Supplementary Data 30: Mouse immune cell type partitioning heritability enrichment for AUD

GWAS using ImmGen gene expression data.
Supplementary Data 31: Epigenetic partitioning heritability enrichment for AUDIT-C GWAS using Roadmap data.
Supplementary Data 32: Epigenetic partitioning heritability enrichment for AUD GWAS using Roadmap data.
Supplementary Data 33: Genetic correlations between different samples in different populations.
Supplementary Data 34: Genetic correlations between AUDIT-C and other traits.
Supplementary Data 35: Genetic correlations between AUD and other traits.
Supplementary Data 36: Test of differences between genetic correlations with AUDIT-C and AUD.
Supplementary Data 37: Genetic correlations between AUDIT-C (AUD adjusted) and other traits.
Supplementary Data 38: Genetic correlations between AUD (AUDIT-C adjusted) and other traits.
Supplementary Data 39: Genetic correlations between AUDIT-C (BMI adjusted) and other traits.
Supplementary Data 40: Genetic correlations between AUD (BMI adjusted) and other traits.
Supplementary Data 41: Prior GWAS associations for GWS AUDIT-C and AUD SNPs.
Supplementary Data 42: Association of polygenic risk scores for AUDIC-T in MVP hold-out sample.
Supplementary Data 43: Association of polygenic risk scores for AUD in MVP hold-out sample.
Supplementary Data 44: Association of polygenic risk scores for AUDIT-C in the Penn Medicine BioBank.
Supplementary Data 45: Association of polygenic risk scores for AUD in the Penn Medicine BioBank.
Supplementary Data 46: Association of polygenic risk scores for AUDIC-T in the Yale-Penn.
Supplementary Data 47: Association of polygenic risk scores for AUD in the Yale-Penn.
Supplementary Data 48: Phenome-wide association analysis of AUDIT-C PRS at two p-value thresholds (1E-07 and All SNPs).
Supplementary Data 49: Phenome-wide association analysis of AUD PRS at two p-value thresholds (1E-07 and All SNPs).
