# Supplementary material for: Genome-wide association study of alcohol consumption and use disorder in 274,424 individuals from multiple populations
Source: Nat Commun. 2019 Apr 2;10:1499. doi: 10.1038/s41467-019-09480-8 (PMC6445072; doi:10.1038/s41467-019-09480-8)
Supplement: Supplementary file 53 — Reporting Summary [file 41467_2019_9480_MOESM53_ESM.pdf]

nature research

Corresponding author(s): *Double-blind peer review submissions: write DBPR and your manuscript number here instead of author names.*

Reporting Summary

Nature Research wishes to improve the reproducibility of the work that we publish. This form provides structure for consistency and transparency in reporting. For further information on Nature Research policies, see Authors & Referees and the Editorial Policy Checklist.

Statistical parameters

When statistical analyses are reported, confirm that the following items are present in the relevant location (e.g. figure legend, table legend, main text, or Methods section).

n/a	Confirmed	
☐	☒	The exact sample size (n) for each experimental group/condition, given as a discrete number and unit of measurement
☐	☒	An indication of whether measurements were taken from distinct samples or whether the same sample was measured repeatedly
☐	☒	The statistical test(s) used AND whether they are one- or two-sided<br>*Only common tests should be described solely by name; describe more complex techniques in the Methods section.*
☐	☒	A description of all covariates tested
☐	☒	A description of any assumptions or corrections, such as tests of normality and adjustment for multiple comparisons
☐	☒	A full description of the statistics including central tendency (e.g. means) or other basic estimates (e.g. regression coefficient) AND variation (e.g. standard deviation) or associated estimates of uncertainty (e.g. confidence intervals)
☒	☐	For null hypothesis testing, the test statistic (e.g. F, t, r) with confidence intervals, effect sizes, degrees of freedom and P value noted<br>*Give P values as exact values whenever suitable.*
☒	☐	For Bayesian analysis, information on the choice of priors and Markov chain Monte Carlo settings
☒	☐	For hierarchical and complex designs, identification of the appropriate level for tests and full reporting of outcomes
☒	☐	Estimates of effect sizes (e.g. Cohen's d, Pearson's r), indicating how they were calculated
☒	☐	Clearly defined error bars<br>*State explicitly what error bars represent (e.g. SD, SE, CI)*

Our web collection on statistics for biologists may be useful.

Software and code

Policy information about availability of computer code

Data collection	N/A
Data analysis	All software used in the analyses is described in the manuscript. All are publicly available and links or references are provided in the manuscript

For manuscripts utilizing custom algorithms or software that are central to the research but not yet described in published literature, software must be made available to editors/reviewers upon request. We strongly encourage code deposition in a community repository (e.g. GitHub). See the Nature Research guidelines for submitting code & software for further information.

Data

Policy information about availability of data

All manuscripts must include a data availability statement. This statement should provide the following information, where applicable:

- Accession codes, unique identifiers, or web links for publicly available datasets
- A list of figures that have associated raw data
- A description of any restrictions on data availability

The dbGaP accession assigned to the Million Veteran Program is phs001672.v1.p. The website at which the data will be made publicly available is: https://www.ncbi.nlm.nih.gov/projects/gap/cgi-bin/study.cgi?study_id=phs001672.v1.p1

Field-specific reporting

Please select the best fit for your research. If you are not sure, read the appropriate sections before making your selection.

☒ Life sciences ☐ Behavioural & social sciences ☐ Ecological, evolutionary & environmental sciences

For a reference copy of the document with all sections, see nature.com/authors/policies/ReportingSummary-flat.pdf

Life sciences study design

All studies must disclose on these points even when the disclosure is negative.

Sample size	We used a series of standard quality control methods to yield a total N = 274,424 for analysis.
Data exclusions	Subjects with genotype call rate < 0.9 or high heterozygosity were removed. Subjects with no demographic information or whose genotypic and phenotypic sex did not match were removed. We also removed one subject randomly from each pair of related individuals. To differentiate population groups, we performed PCA analyses and participants with PC scores > 3 standard deviations from the mean of any of the 10 PCs were removed as outliers. Finally, individuals < 22 or > 90 years old and those with missing AUDIT-C scores were removed.
Replication	Population-specific summary statistics from the AUDIT-C and AUD GWAS in MVP were used to generate polygenic risk scores in the Penn Medicine Biobank, an independent sample of EAs (N = 7,420) and AAs (N = 2,031).
Randomization	Not applicable
Blinding	Genotyping was done blind to phenotype

Reporting for specific materials, systems and methods

Materials & experimental systems

n/a	Involved in the study	
☒	☐	Unique biological materials
☒	☐	Antibodies
☒	☐	Eukaryotic cell lines
☒	☐	Palaeontology
☒	☐	Animals and other organisms
☐	☒	Human research participants

Methods

n/a	Involved in the study	
☒	☐	ChIP-seq
☒	☐	Flow cytometry
☒	☐	MRI-based neuroimaging

Human research participants

Policy information about studies involving human research participants

Population characteristics	We used principal components analysis and data from the 1000 Genomes sample to differentiate five population groups: European Americans, African Americans, Latino Americans, East Asian Americans, and South Asian Americans.
Recruitment	Participants were recruited through the U.S. Veterans Administration (VA) Million Veteran Program, which advertised and solicited patients receiving medical care through the VA. They gave informed consent for use of their self-report information and access to their electronic medical record. They also provided a blood sample for DNA extraction and genotyping.
